# Bacterial and host interactions in *Staphylococcus aureus*-induced septic arthritis—understanding pathogenesis and exploring therapeutic strategies

**DOI:** 10.3389/fmicb.2025.1687243

**Published:** 2025-10-15

**Authors:** Zhicheng Hu, Cecilia Engdahl, Rille Pullerits, Majd Mohammad, Tao Jin

**Affiliations:** ^1^Center for Clinical Laboratories, The Affiliated Hospital of Guizhou Medical University, Guiyang, China; ^2^Department of Rheumatology and Inflammation Research, Institute of Medicine, The Sahlgrenska Academy, University of Gothenburg, Gothenburg, Sweden; ^3^Department of Clinical Immunology and Transfusion Medicine, Sahlgrenska University Hospital, Gothenburg, Sweden; ^4^Department of Rheumatology, Sahlgrenska University Hospital, Gothenburg, Sweden

**Keywords:** *Staphylococcus aureus*, septic arthritis, virulence factors, immunity, osteoimmunology, antibiotic resistance, bacteriophage therapy, vaccines

## Abstract

Septic arthritis is a severe and rapidly destructive joint infection, primarily caused by *Staphylococcus aureus*. The interplay between bacterial virulence factors and host immune responses determines disease progression and clinical outcomes. This review discusses the key bacterial factors that contribute to septic arthritis, including *S. aureus* cell wall components, surface proteins, and secreted toxins. In parallel, host-related factors, such as aging, immune responses, and genetic predispositions, are examined in conjunction with the impact of *S. aureus* infection on bone integrity and osteoimmunological mechanisms. Finally, this review highlights emerging therapeutic approaches, including targeted anti-virulence strategies, immune modulation, and anti-osteoclastogenic interventions, in mitigating joint damage. Understanding the multifaceted interactions between *S. aureus* and the host immune system is crucial for advancing treatment strategies and reducing morbidity associated with septic arthritis.

## 1 Introduction

Septic arthritis, recognized as one of the most aggressive joint diseases, is characterized by inflammation, rapid cartilage degradation, and bone destruction (Nguyen et al., 2023; Goldenberg, 1998). Despite prompt therapeutic intervention, approximately 50% of patients experience progression to irreversible structural joint damage, culminating in chronic disability characterized by lifelong functional impairment (Mohammad et al., 2019). Nine percent of patients previously affected by septic arthritis underwent arthroplasty within 15 years, indicating a risk that is six-fold higher than that of the general population (Abram et al., 2020). Septic arthritis primarily occurs via hematogenous dissemination, wherein pathogens enter the systemic blood circulation, colonize the joint synovial membrane, and ultimately establish infection within the joint cavity. Secondary routes include direct inoculation through traumatic injuries, iatrogenic procedures (e.g., arthrocentesis), or contiguous extension from adjacent osteomyelitic foci (Mathews et al., 2010). The infection incites a dysregulated innate immune cascade marked by massive neutrophil infiltration, monocyte/macrophage activation, and excessive release of pro-inflammatory mediators and proteolytic enzymes (Garcia-Arias et al., 2011; Momodu and Savaliya, 2024). This inflammatory milieu drives synovial hyperproliferation, cartilage matrix degradation, and bone resorption, ultimately leading to irreversible joint destruction if left untreated. Notably, even following timely antibiotic administration, residual bacterial components, such as bacterial DNA and lipoproteins (Lpps), persist as immunostimulatory pathogen-associated molecular patterns (PAMPs), perpetuating osteolytic lesions via receptor activator of nuclear factor-κB (NF-κB) ligand (RANKL)-mediated osteoclastogenesis (Mohammad et al., 2019, 2022; Ali et al., 2015b; Hu et al., 2025). Recent epidemiological studies estimate septic arthritis incidence in Western countries at 2–10 cases per 100,000 person-years (Tarkowski, 2006), with a bimodal age distribution showing elevated susceptibility among neonates/children aged 2–3 years and individuals >80 (Hu et al., 2023). High-risk cohorts include prosthetic joint recipients, immunocompromised hosts (e.g., diabetes and HIV), and patients with pre-existing inflammatory arthropathies [e.g., rheumatoid arthritis (RA)] (Tarkowski, 2006; Nade, 2003; Colavite and Sartori, 2014; Jin et al., 2021), with mortality rates exceeding 15% in patients having multiple comorbidities such as diabetes or chronic kidney disease (Schindler et al., 2025).

The diagnosis of septic arthritis is often considered straightforward, with synovial fluid (SF) culture serving as the gold standard for diagnosis. However, this method has limitations: only approximately 50% of patients yield a positive culture result (Alexandersson et al., 2025), and cultures require time to process. To address this, molecular methods, such as polymerase chain reaction (PCR) assays, have been investigated on SF. Although studies indicate that PCR does not improve diagnostic accuracy over culture for common pathogens such as *Staphylococcus aureus* and streptococci, it may offer advantages in identifying less common pathogens such as *Borrelia* species (Mathews and Coakley, 2008).

In addition to microbiological testing, several laboratory parameters contribute to diagnostic evaluation. Although blood tests are commonly performed, the most valuable information comes from SF analysis. Classic diagnostic cutoffs, established by Ropes and Bauer in the 1950s, remain widely used: SF white blood cell (WBC) count > 50,000 cells/mm3, serum/SF glucose ratio < 0.5, and polymorphonuclear percentage (PMN%) > 90 (Ropes and Bauer, 1953). Notably, the summary likelihood ratio increases progressively with higher SF WBC counts and PMN% (Margaretten et al., 2007). However, growing evidence suggests that rigid adherence to these classic cutoffs may lack sensitivity, particularly in certain patient populations or clinical scenarios (Streck et al., 2025). This underscores the need for integrating multiple diagnostic parameters; importantly, while these classical cutoffs primarily apply to native joint infections, lower thresholds are used for periprosthetic joint infection (PJI; Parvizi et al., 2018; Xu et al., 2019).

Septic arthritis should be treated with prompt antimicrobial therapy, which should be initiated based on clinical suspicion, even before SF or blood culture results are available. The choice of antibiotics is usually empirical, guided by the presence of risk factors for atypical organisms (Mathews and Coakley, 2008). The recommended duration is typically up to 2 weeks of intravenous therapy or until clinical improvement is evident, followed by 4 weeks of oral antibiotics (Coakley et al., 2006). In addition, expert consensus emphasizes the essential role of urgent source control, which involves evacuating purulent material from the joint space. This can be achieved via closed needle aspiration, arthroscopic lavage, or open debridement (Coakley et al., 2006). This is not unexpected, as bacterial components are known to provoke intense proinflammatory responses and contribute significantly to joint destruction, as discussed in the later section.

Outcomes of septic arthritis remain concerning. Mortality is relatively high, ranging from 10 to 30%, and can lead to an even higher rate in cases of polyarthritis (Weston et al., 1999; Kaandorp et al., 1997; Jung et al., 2018). Osteomyelitis develops in approximately 8% of patients (Weston et al., 1999). Subjectively poor joint outcomes have been reported in 20–30% of cases (Kaandorp et al., 1997; Ferrand et al., 2016). A recent nationwide study conducted in the UK, which included all patients undergoing arthroscopic knee washout over 20 years, revealed a 90-day mortality rate of 8.94%, with risk of death increasing with age (odds ratio per 5-year increase: 1.38). Notably, 8.76% of patients underwent arthroplasty within 15 years, indicating a six-fold higher risk compared to the general population (Abram et al., 2020). Interestingly, the risk of arthroplasty was significantly higher in patients with a history of osteoarthritis or RA than in those without prior joint disease (Abram et al., 2020).

**Figure 1:**
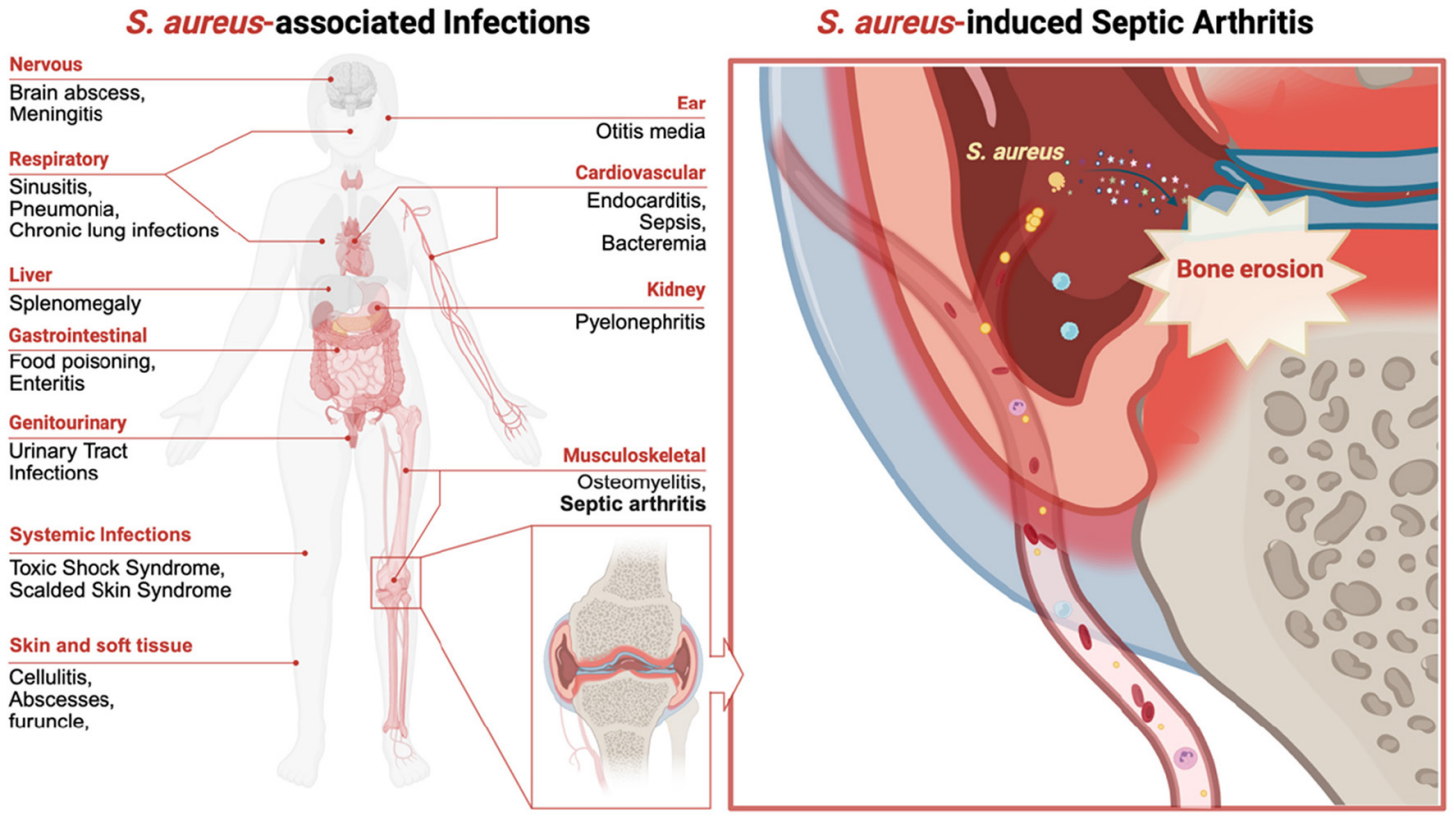
Schematic diagram illustrating the clinical spectrum of *Staphylococcus aureus* and the pathogenesis of septic arthritis. This figure illustrates the various infections caused by *S. aureus*, which affect the nervous, respiratory, cardiovascular, musculoskeletal, gastrointestinal, genitourinary, and skin and soft tissue systems. The left panel highlights diseases such as sepsis, bacteremia, pneumonia, endocarditis, osteomyelitis, and toxic shock syndrome. The right panel depicts an *S. aureus*-induced septic arthritis knee joint with greater magnification, illustrating bacterial adhesion, invasion, biofilm formation, and the resulting joint damage.

Septic arthritis arises from a diverse microbial etiology, with *S. aureus* emerging as the predominant etiological agent, accounting for the majority of clinically diagnosed cases (Goldenberg, 1998; Alexandersson et al., 2025; Jin, 2024). This pathogen exhibits broad clinical pathogenicity, spanning from localized cutaneous infections to systemic, life-threatening conditions, including brain abscess, meningitis, pneumonia, endocarditis, sepsis, bacteremia, toxin-mediated gastroenteritis, toxic shock syndrome (TSS), and osteomyelitis (as illustrated in Figure 1).

The virulence of *S. aureus* is attributed to its ability to evade host immune responses, produce cytotoxic molecules, and establish biofilms that contribute to chronic infections (Mohammad et al., 2022; Na et al., 2020). A retrospective cohort study published in *The Lancet* demonstrated that, among 33 analyzed bacterial pathogens, *S. aureus* was the sole pathogen responsible for exceeding 1.1 million attributable deaths globally (Collaborators, 2022). The pathogenesis and clinical expression of *S. aureus* infections are modulated by complex host–pathogen interactions involving microbial virulence factors and host immunological defenses.

**Figure 2:**
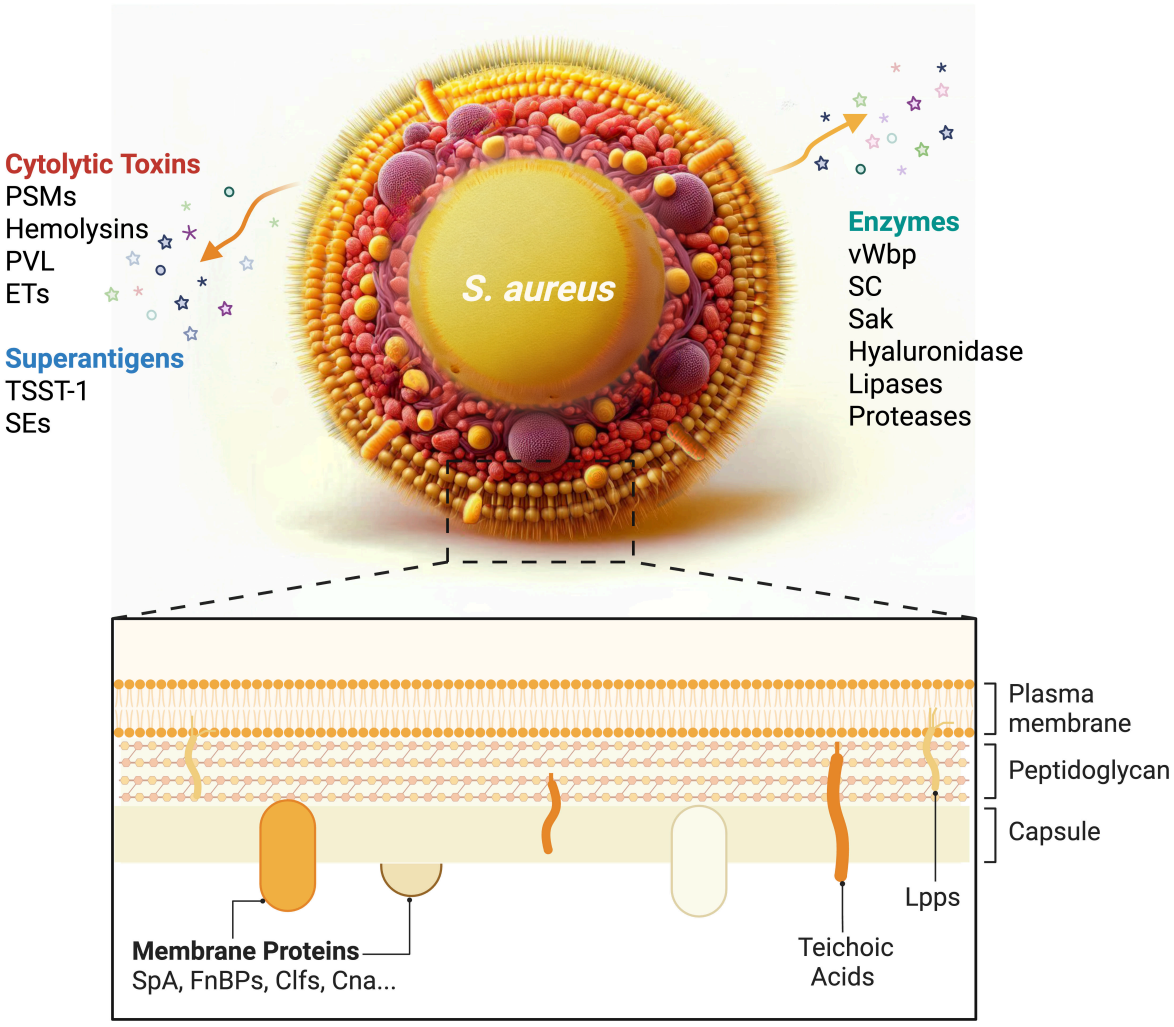
Schematic illustration depicting the structural organization of Staphylococcus aureus and its virulence factors.

This comprehensive review systematically examines key bacterial mediators implicated in septic arthritis pathogenesis, encompassing *S. aureus* structural components (e.g., peptidoglycan, teichoic acids, and lipoproteins), secreted exotoxins (e.g., α-hemolysin and Panton–Valentine leukocidin), and immunoevasive molecules (e.g., protein A and chemotaxis inhibitory protein) as described below and illustrated in Figure 2. Concurrently, host-related variables influencing susceptibility and disease severity are discussed, including age-associated immunosenescence, dysregulated cytokine cascades, and polymorphisms in innate immune receptors, such as Toll-like receptors (TLRs) and formyl peptide receptors (FPRs). Pathophysiological consequences of *S. aureus* infection on osseous integrity are critically evaluated, with an emphasis on osteoclast activation, the RANKL/osteoprotegerin (OPG), axis perturbation, and osteoblast dysfunction as demonstrated below. Furthermore, emerging translational interventions are highlighted, including precision anti-virulence therapeutics (e.g., quorum-sensing inhibitors), immunomodulatory biologics, phage-derived lysins, and multivalent vaccine candidates targeting adhesins and toxoids.

## 2 *S. aureus*—bacterial factors

The formidable virulence potential of *S. aureus* stems from its structurally complex cell wall components, such as peptidoglycan and lipoteichoic acids, combined with a multifactorial arsenal of virulence determinants. These include pore-forming exotoxins (e.g., α-hemolysin and Panton–Valentine leukocidin), immune–evasion molecules (e.g., protein A and staphylococcal superantigen-like proteins), and extracellular enzymes (e.g., proteases and coagulases), which collectively mediate tissue invasion, immune subversion, and host damage, as described in more detail below and delineated in Figure 2 and Table 1.

**Table 1:** Key virulence molecules expressed by *Staphylococcus aureus*.

**Molecules**	**Functions**	**Arthritogenic roles**
**Secreted molecules**		
vWbp, SC	Binds fibrinogen, triggering clot formation to shield bacteria.	Facilitate joint invasion and exacerbate bone erosion (Na et al., 2020; Friedrich et al., 2003).
Sak	Activates plasminogen → plasmin, degrading fibrin clots to facilitate hematogenous dissemination.	Attenuates sepsis via plasminogen activation (Kwieciński et al., 2010).
SCIN	Inhibits complement activation (Koymans et al., 2017; Rooijakkers et al., 2006).	Exacerbates disease severity (Sakiniene et al., 1999).
Thermonuclease A	Increases the extracellular and intracellular survival of bacteria.	Exacerbates septic arthritis (Li et al., 2025).
**Cell wall components**		
Lpps	Virulence factor expression, nutrient uptake, antibiotic resistance, and host–pathogen interplay (Mohammad et al., 2022).	Potent proinflammatory effects in systemic, localized, and cutaneous infections (Mohammad et al., 2019, 2020, 2021).
PGN	Critical bacterial cell wall structural polymer.	Limited effect in inducing synovitis (Mohammad et al., 2019).
SpA	Binds IgG Fc → blocks phagocytosis and opsonization (Cruz et al., 2022).	Exacerbating disease severity and outcomes (Palmqvist et al., 2002).
FnBPs	Facilitate bacterial adhesion.	Limited effect in arthritogenicity (Palmqvist et al., 2005; Speziale and Pietrocola, 2020).
ClfA and ClfB	Promote bacterial adhesion.	Involved in osteoclastogenesis and cartilage degradation (Palmqvist et al., 2005).
Cna	Enable bacterial colonization in joint cartilage (Switalski et al., 1993).	Exacerbates disease severity and outcomes (Patti et al., 1994).
Surface proteins (e.g., FnBPs and ClfA/B)	Contribute to biofilm development and tissue adhesion.	Amplify inflammasome activation and drive osteoclastogenesis and cartilage degradation (Palmqvist et al., 2005).
CPs	Contribute to immune evasion and enhance bacterial virulence.	Exacerbate arthritic and septic outcomes (Ko et al., 2013; Nilsson et al., 1997).
**Toxins and other molecules**		
PSMs	Contribute to biofilm formation, cell lysis and weaken immune response (Cheung et al., 2014; Peschel and Otto, 2013).	PSMα aggravates systemic infection, but PSMβ protects against joint destruction (Hu et al., 2022).
α-Toxin	Contributes to cell lysis and tissue damage, and immune suppression (Weiss et al., 2023).	Exacerbates the frequency and severity of arthritis (Jarneborn et al., 2020).
Bacterial DNA	Induce inflammatory response (Mann et al., 2009; Sharma and Rajpurohit, 2024; Fournier and Philpott, 2005).	Exacerbates outcomes of arthritis and septic shock (Deng et al., 1999; Sparwasser et al., 1997).

### 2.1 Cell wall components

#### 2.1.1 Peptidoglycan (PGN)

PGN, a critical structural polymer of the staphylococcal cell wall, confers mechanical stability and osmotic resistance through its three-dimensional mesh-like exoskeleton. Composed of repeating glycan chains of β-(1,4)-linked N-acetylglucosamine (NAG) and N-acetylmuramic acid (NAM) residues cross-linked by short peptide bridges, PGN forms a rigid outer layer that maintains cell shape and integrity (Sutton et al., 2021).

Fragments of PGN can function as PAMPs recognized by pattern recognition receptors (PRRs), specifically via nucleotide-binding oligomerization domain-containing protein 2 (NOD2) in macrophages and dendritic cells. This recognition initiates pro-inflammatory signaling cascades [e.g., NF-κB and mitogen-activated protein kinase (MAPK)] that drive antimicrobial peptide synthesis and inflammasome activation (Reed et al., 2015).

Contradictory evidence exists regarding PGN's arthritogenic potential. Early studies reported that intra-articular administration of purified staphylococcal PGN in murine models induced severe arthritis marked by robust macrophage/neutrophil infiltration, synovial hyperplasia, and bone erosion (Liu et al., 2001). In contrast, our recent findings demonstrate that PGN isolated from an *S. aureus lgt* mutant strain (deficient in Lpps biosynthesis) elicited only transient, mild synovitis without significant joint destruction (Mohammad et al., 2019). This observed variability likely reflects strain-specific differences in PGN biochemical composition and purity, as traditional purification methods may co-isolate immunostimulatory Lpps, confounding earlier results.

#### 2.1.2 Lipoproteins (Lpps)

Staphylococcal Lpps, fundamental to bacterial membrane architecture, play multifaceted roles in virulence factor expression, nutrient uptake, antibiotic resistance, and host–pathogen interplay (Mohammad et al., 2022). These lipid-anchored proteins function as potent Toll-like receptor 2 (TLR2) agonists, driving pro-inflammatory cascades in macrophages and dendritic cells that eventually lead to tissue damage. Lpps have been systematically characterized by our research group across multiple murine disease models, including septic arthritis, sepsis, and cutaneous infection, revealing context-dependent roles shaped by infection route and target tissue.

Systemic infection (intravenous administration): Lpps enhance bacterial metabolic fitness via nutrient scavenging and immune evasion, acting as critical virulence determinants. TLR2/MyD88-dependent signaling exacerbates systemic inflammation, manifesting as accelerated weight loss, impaired bacterial clearance, and increased mortality in murine models (Mohammad et al., 2020).

Localized joint infection (intra-articular administration): Lpps exhibit an immunomodulatory double-edged effect: Purified Lpps alone induce severe bone erosion, whereas co-injection of purified Lpps with live *S. aureus* exhibits adjuvant-like properties, thereby enhancing bacterial killing and attenuating joint destruction, highlighting a protective role in septic arthritis (Mohammad et al., 2019).

Cutaneous infection (subcutaneous administration): Lpps amplify the local inflammatory response through leukocyte chemoattractants and myeloperoxidase, correlating with elevated bacterial persistence. Concurrently, Lpps drive dysregulated hemostasis, fostering fibrin-rich abscess formation that physically shields bacteria from leukocyte infiltration (Mohammad et al., 2021).

#### 2.1.3 Surface proteins

*S. aureus* employs a diversity of surface proteins covalently secured to its cell wall via sortase-catalyzed LPXTG motif anchoring, which contribute to critical pathogenic processes including host adhesion, immune evasion, and tissue invasion. These virulence determinants are categorized as Microbial Surface Components Recognizing Adhesive Matrix Molecules (MSCRAMMs). These proteins bind to host regulatory components, effectively hijacking endogenous “off-switches” of the complement cascade and enabling ligand-specific interactions with host extracellular matrix components and cellular receptors, particularly within synovial and chondrocytic niches (Foster, 2005; Foster et al., 2014; Kim et al., 2017). Key MSCRAMMs implicated in the pathogenesis of septic arthritis include:

##### 2.1.3.1 Staphylococcal protein A (SpA)

SpA binds the Fcγ domain of immunoglobulins (IgG), disrupting opsonophagocytosis by masking bacterial surfaces from antibody-dependent clearance (Cruz et al., 2022). SpA-IgG immune complexes further trigger TNFR1-dependent apoptosis in B lymphocytes and cause monocytes to undergo necrosis (Fox et al., 2021).

In a murine septic arthritis model, wild-type *S. aureus* (Newman strain) induced significantly higher synovial tumor necrosis factor (TNF)-α levels, severe arthritis, and increased mortality compared to isogenic *spa* mutants, demonstrating SpA functions as a critical virulence factor responsible for exacerbating disease severity and outcomes in septic arthritis (Palmqvist et al., 2002).

##### 2.1.3.2 Fibronectin-binding proteins (FnBPs)

FnBPs mediate high-affinity binding to fibronectin's N-terminal domain via a tandem β-zipper mechanism, facilitating bacterial attachment to endothelial cells and articular tissues.

While FnBPs exhibit negligible direct arthritogenicity, they drive interleukin-6 (IL-6)-dominated cytokine storms via integrin α5β1/fibronectin bridging, thereby inducing weight loss, increased mortality, and bacteremia in murine sepsis models (Palmqvist et al., 2005; Speziale and Pietrocola, 2020). This systemic inflammation may indirectly potentiate joint vulnerability to metastatic infection.

##### 2.1.3.3 Clumping factors (ClfA and ClfB)

ClfA/B bind fibrinogen's γ-chain via a “dock, lock, and latch” mechanism mediated by their DEv-IgG-fold domains, promoting adhesion to fibrin deposits in inflamed synovium (Jin, 2024; Deivanayagam et al., 2002).

In murine models, ClfA/B synergize with FnBPs to amplify NLRP3 inflammasome activation in synovial macrophages, driving IL-1β-mediated osteoclastogenesis and cartilage degradation. Dual *clfA/clfB* deletion reduced arthritis incidence by 60% compared to infections induced by its wild-type counterpart (Palmqvist et al., 2005). The severity of septic arthritis was markedly reduced in mice infected with a ClfA mutant but not a ClfB mutant, compared with mice infected with the wild-type strain. In fact, ClfA vaccination prevented septic arthritis, suggesting that ClfA is crucial for septic arthritis development (Josefsson et al., 2001).

##### 2.1.3.4 Collagen adhesion (Cna)

Cna's collagen-binding domains (CBDs) bind type I/II collagen triple helices via a unique “collagen hug” mechanism, enabling *S. aureus* colonization of articular cartilage (Switalski et al., 1993).

It has been revealed that the collagen-binding protein (CnBP) was detectable in 56% of *S. aureus* isolates associated with osteomyelitis, underscoring its pathophysiological relevance to bone and collagen matrix colonization (Ryding et al., 1997). In murine infection models, animals inoculated with a *cnbP*-deficient isogenic mutant exhibited a markedly reduced incidence of septic arthritis (27% vs. 70%), indicating that CnBP is a critical determinant of arthritogenic virulence and disease progression (Patti et al., 1994).

#### 2.1.4 Capsular polysaccharides (CPs)

*S. aureus* synthesizes several capsular polysaccharides (CPs), aiding in immune evasion and enhancing virulence, primarily serotypes 5 (CP5) and 8 (CP8), which dominate clinical isolates (Ryding et al., 1997; Arbeit et al., 1984).

These exopolysaccharides form an anti-phagocytic shield by sterically hindering opsonin deposition and masking surface epitopes from antibody recognition (Ko et al., 2013). CP5, in particular, exacerbates arthritic and septic outcomes: murine models infected with CP5-expressing strains exhibit 2-fold higher mortality and 2.3-fold increased arthritis severity compared to Δ*cap5* isogenic mutants (Nilsson et al., 1997).

### 2.2 Secreted molecules

*S. aureus* deploys a series of secreted molecules to subvert host defenses, establish infection, and disseminate disease. These effectors, categorized by functional specialization (Table 1), include enzymatic toxins, immune modulators, and biofilm-associated molecules.

#### 2.2.1 von Willebrand factor-binding protein (vWbp)

vWbp is a hemostatic hijacker that binds von Willebrand factor (vWF), a glycoprotein critical for platelet adhesion and clot stabilization. By anchoring to endothelial vWF multimers, vWbp facilitates *S. aureus* adhesion to synovial microvasculature, enabling joint invasion and immune evasion via fibrin-encapsulated microcolonies (Thomas et al., 2021; Drakeford et al., 2022).

In murine septic arthritis models, Δvwb mutants exhibited three-fold reduced joint bacterial loads compared to wild-type strains, while vWF-deficient mice infected with the Δvwb (vwb-mutant) strain exhibited more severe bone erosion, underscoring vWbp's capacity for joint invasion and the role in joint-specific pathogenicity (Na et al., 2020). Importantly, no difference in arthritis severity was found between Δvwb mutants and the wild-type strain in vWF-deficient mice, suggesting the arthritogenic effect of vWbp might be mediated by vWbp-vWF complex formation. RA is known to be the major risk factor for septic arthritis (Favero et al., 2008). In RA, inflammatory cytokines stimulate endothelial cells to release extra-large and hyperreactive vWF multimers (Bernardo et al., 2004). We propose that hyperactive vWbp may exploit these vWF multimers to generate microthrombus-like niches within the synovial microvasculature, thereby facilitating joint invasion by *S. aureus* expressing vWbp in RA patients. It is known that vWbp can interact with ClfA on the bacterial surface after secretion, promoting *S. aureus* adhesion to vWF and vascular endothelium under shear stress (Claes et al., 2017). However, whether the arthritogenic properties of vWbp are mediated through ClfA remains unknown, warranting further studies.

#### 2.2.2 Staphylocoagulase (SC)

SC and vWbp share 78% sequence homology and both activate prothrombin to convert fibrinogen to fibrin, fostering abscess formation (Friedrich et al., 2003). Furthermore, SC-positive *S. aureus* strains possess molecular strategies to resist fibrin clot-mediated clearance, facilitating bacterial aggregation, enhancing intraclot survival, and sustaining chronic host colonization, thereby potentiating virulence (Loof et al., 2015).

However, SC's contribution to septic arthritis is auxiliary: SC deficient (Δ*coa*) strains showed only 28% attenuation in synovitis severity compared to wild-type, contrasting with Δ*vwb*'s 69% reduction, indicating the role of SC in joint invasion is less significant compared to vWbp (Na et al., 2020).

#### 2.2.3 Staphylokinase (Sak)

Sak, an agr-regulated fibrinolytic enzyme, activates plasminogen to generate plasmin, thereby dissolving fibrin clots and facilitating hematogenous dissemination. Paradoxically, Sak also challenges innate immunity: Sak-mediated plasmin proteolysis degrades biofilm matrices, sensitizing *S. aureus* to antibiotics and immune defenses (Kwiecinski et al., 2016). Sak-α-defensin complexes neutralize neutrophil extracellular trap (NET) bactericidal activity, protecting against α-defensin-mediated killing (Jin et al., 2004).

Sak expression had no impact on disease development in the mouse septic arthritis model. However, Sak attenuates sepsis via plasminogen activation since Δsak caused higher mortality vs. wild-type bacteria (Kwieciński et al., 2010). Contrariwise, in cutaneous infection, Sak promotes abscess drainage, reducing lesion size (Kwiecinski et al., 2013).

#### 2.2.4 Staphylococcal complement inhibitor (SCIN)

SCIN blocks complement-mediated opsonophagocytosis by stabilizing C3 convertase, preventing amplification, and inhibiting C5a anaphylatoxin generation, reducing neutrophil chemotaxis (Rooijakkers et al., 2007).

In murine models of *S. aureus*-induced septic arthritis and bacteremia, complement depletion (via cobra venom factor) exacerbated disease severity, due to dysregulated innate immunity characterized by compromised neutrophil chemotaxis and transendothelial migration, as well as defective opsonophagocytosis (Sakiniene et al., 1999). The role of SCIN in the development of septic arthritis is still unclear.

#### 2.2.5 Thermonuclease A (NucA)

NucA, a nuclease produced by *S. aureus*, degrades extracellular DNA and RNA, which destabilizes biofilms and causes bacterial dispersal (Bhattacharya et al., 2020). Loss of NucA (Δ*nuc1* mutant) results in stronger biofilm formation, more neutrophil extracellular trap (NETs) production, and increased bacterial killing by neutrophils (Berends et al., 2010). NucA helps *S. aureus* evade NETs by breaking down their DNA backbone and generating nucleoside products, inducing apoptosis in immune cells (Thammavongsa et al., 2013). In infection models, NucA expression is linked to higher mortality, delayed bacterial clearance, and resistance to neutrophil killing (Berends et al., 2010).

In a mouse model of septic arthritis, NucA causes severe bone destruction, rapid weight loss, and high proinflammatory cytokine levels. These effects might be mediated through its NET-degrading activity, suppression of neutrophil killing, and cytokine induction in host cells, making NucA a key driver of *S. aureus* septic arthritis (Li et al., 2025).

### 2.3 Toxins

*S. aureus* produces a variety of toxins that significantly enhance its pathogenicity. In addition to those mentioned above, numerous other toxins contribute to disease progression by damaging host tissues, disrupting immune responses, and promoting bacterial dissemination. These toxins can be grouped based on their functions and targets as follows:

#### 2.3.1 Phenol-soluble modulins (PSMs)

PSMs are a family of small, amphipathic peptides that play crucial roles in the virulence of *S. aureus*. PSMα, particularly α3, is described as highly toxic and contributes to the pathogenicity of *S. aureus* by lysing host neutrophils and other immune cells, leading to the release of enzymes and reactive oxygen species (ROS) into the surrounding tissue. This process can cause inflammation, tissue damage, and ultimately result in organ dysfunction while also weakening the immune response (Cheung et al., 2014). While PSMα has been considered as a potent cytolysin, all *S. aureus* PSMs play a role in biofilm formation (Cheung et al., 2014; Peschel and Otto, 2013). Especially, the aggregation of PSMα3 into amyloid fibril fortifies the biofilm structure, enhances its resistance to mechanical stress and matrix-degrading enzymes (Peschel and Otto, 2013; Schwartz et al., 2012).

Recent research investigated the roles of PSMα and PSMβ using a mouse *S. aureus* septic arthritis model with three isogenic *S. aureus* strains: Newman (wild type), Δ*psm*α (PSMα-deficient), and Δ*psm*β (PSMβ-deficient; Hu et al., 2022). This study concludes that PSMα and PSMβ have distinct roles in septic arthritis: PSMα aggravates systemic infection but does not significantly impact the development of septic arthritis. However, Δ*psm*β-infected mice showed increased bone erosion in septic arthritis, indicating that PSMβ protects against joint destruction. These findings highlight the complex interplay between different PSMs in the pathogenesis of staphylococcal infections and suggest potential therapeutic targets for managing septic arthritis (Hu et al., 2022).

#### 2.3.2 α-Toxin (alpha-hemolysin)

Among all the hemolysins, α-toxin is the most studied molecule due to its potent virulence. It is known as a pore-forming toxin, which creates transmembrane channels in host cell membranes, including red blood cells, epithelial cells, and immune cells, leading to cell lysis and tissue damage, as well as immune suppression (Krones et al., 2021). It has been confirmed to contribute to the pathogenesis of many different diseases, including septic arthritis and sepsis.

In a mouse model, infection with an alpha-toxin mutant strain of *S. aureus* resulted in a lower frequency and reduced severity of arthritis (Nilsson et al., 1999). Similarly, a rabbit sepsis model demonstrated that rabbits infected with the alpha-toxin mutant strain showed no mortality, whereas half of the control group succumbed to the infection by day 10 (Crémieux et al., 2014).

#### 2.3.3 Superantigens

*S. aureus* superantigens have the capability of activating a significant portion (up to 20%) of host T cells and causing the release of inflammatory cytokines, leading to the pathogenesis of life-threatening infections like toxic shock syndrome (TSS), food poisoning, and septic shock (Noli Truant et al., 2022; Roetzer et al., 2022). They can be categorized into three types: Toxic shock syndrome toxin-1 (TSST-1), staphylococcal enterotoxins (SEs), and staphylococcal enterotoxin-like toxins (SEIs). TSST-1 is critical in the pathogenesis of toxic shock syndrome (TSS), leading to severe complications such as multiple organ disorder and shock (Weiss et al., 2023). One of our previous studies indicated that tofacitinib, an immunosuppressive medicine for RA, significantly reduced mortality, accompanied by decreased levels of interferon γ (IFN-γ) and TNF-α, in mouse models of TSST-1 induced shock, which highlights the protective effect of tofacitinib in enterotoxin-induced shock in mice (Jarneborn et al., 2020).

In both rat and mouse models for septic arthritis, superantigens such as TSST-1 were shown to play a potent role, as TSST-1-deficient strain-infected mice developed less severe and less frequent septic arthritis compared to mice infected with the TSST-1 parental strain (Bremell and Tarkowski, 1995; Abdelnour et al., 1994a).

The roles of other virulence factors in septic arthritis are still under investigation, including chemotaxis inhibitory protein of staphylococci (CHIPS), wall teichoic acids (WTAs), lipoteichoic acids (LTAs), polysaccharide intercellular adhesin (PIA), hyaluronidase, lipases, and proteases. Understanding these virulence factors is crucial for developing new therapeutic strategies to combat *S. aureus* infections, particularly in the face of rising antibiotic resistance.

## 3 Host factors

Host factors play a very important role in *S. aureus* septic arthritis. The susceptibility, progression, and prognosis of the disease are greatly dependent on the host's age, immune response, gender, hormonal changes, genetic susceptibility, and other factors, as reviewed below.

### 3.1 Aging

Aging is an important factor influencing the host's susceptibility to septic arthritis and the severity of the disease. Advanced age (over 80 years) was identified as a significant risk factor for septic arthritis in a prospective cohort study in Amsterdam, which monitored 4,907 patients with rheumatic diseases for 3 years and identified 37 new cases of septic arthritis (Kaandorp et al., 1995). Indeed, our data suggest that the incidence of septic arthritis is six times higher in persons over 80 compared to those under 65 (Alexandersson et al., 2024). The immune system and physiological functions of the human body undergo significant changes with age. These changes not only increase the risk of infection in the elderly but also aggravate the consequences of infection. Thus, it is critical to understand how aging influences the host immune response and the progression of the disease.

#### 3.1.1 Immunosenescence

Immunosenescence refers to the gradual decline in immune function and changes in immune response that occur in the elderly, encompassing both innate and adaptive immunity. Innate immunity in the elderly is characterized by a decline in the function of neutrophils, macrophages, and natural killer (NK) cells, and these cells play an important role in fighting bacterial infections (Arlt and Hewison, 2004). Neutrophils exhibit a weakened trend of chemotaxis and phagocytosis, while macrophages show a reduction of antigen presentation ability and phagocytosis function (Plowden et al., 2004; Simmons et al., 2022), facilitating bacterial colonization and proliferation in the host.

In adaptive immunity, the population and function of T cells and B cells are affected. Due to aging, the thymus of the elderly gradually degenerates, resulting in a decrease in the production of new T cells, affecting the diversity and function of existing T cells (Srinivasan et al., 2023; Snijckers and Foks, 2024). In addition, the production of B cells and the ability to produce antibodies have also been weakened, which causes an insufficient immune response to new infections in the elderly population (Snijckers and Foks, 2024). Therefore, these alterations contribute to immunosenescence, making the aged immune system more susceptible and resulting in a lower clearance efficiency when dealing with pathogens, such as *S. aureus*.

In a mouse bacteremia model, we found that advanced age increased mortality, altered splenomegaly, and was associated with impaired cytokine responses and dysfunctional myeloid cell activity despite elevated circulating neutrophils and monocytes (Hu et al., 2023; Nacionales et al., 2014). However, the frequency and severity of septic arthritis did not differ with age, despite prior evidence of compromised immunoglobulin responses in older mice after *S. aureus* infection (Gupta et al., 2023).

#### 3.1.2 Changes of microenvironment in the joint

The joints themselves also undergo a significant intrinsic decline with age. These changes include the wear and tear of cartilage, the increased inflammatory response of the synovium, and the changes in the composition of the synovial fluid (Loeser, 2010). These degenerative changes not only make the elderly more susceptible to chronic joint diseases such as osteoarthritis but also provide favorable conditions for the occurrence of septic arthritis.

The level of immunomodulatory factors in joint synovial fluid changes with age, impacting the local anti-infection ability (Muire et al., 2020; Lencel and Magne, 2011). Aging-related reductions in key components, such as hyaluronic acid and lubricating proteins, in synovial fluid compromise the lubricating and cushioning functions of the joints, potentially diminishing their barrier function against invasive bacteria (Temple-Wong et al., 2016). In addition, in elderly individuals, reduced blood flow in synovial tissues and impaired efficiency of immune cell migration to sites of infection create conducive conditions for *S. aureus* colonization and proliferation within the joints (Sacitharan, 2019).

#### 3.1.3 Chronic diseases

A variety of chronic diseases, like cardiovascular diseases, diabetes, and chronic kidney diseases, are often accompanied by the elderly, which further increases the risk of infection. For example, diabetes significantly increases the susceptibility to bacterial infections due to factors like hyperglycemia-induced immune dysfunction, impaired cytokine production, and altered Sabers two-component system (a regulatory system in *S. aureus* that controls the expression of various virulence factors; Genito et al., 2023; Wang et al., 2023; Zhou et al., 2024). Patients with cardiovascular disease and chronic kidney disease have reduced anti-infection ability due to disorders of blood circulation and metabolic function, as reviewed in Marassi and Fadini (2023) and Zoccali et al. (2023). In addition, the existence of a variety of chronic diseases is often accompanied by long-term use of immunosuppressants, steroids, and other drugs, which can also inhibit the immune system and increase the risk of infection (Zoccali et al., 2023).

### 3.2 *S. aureus*-caused immune response in infection

**Figure 3:**
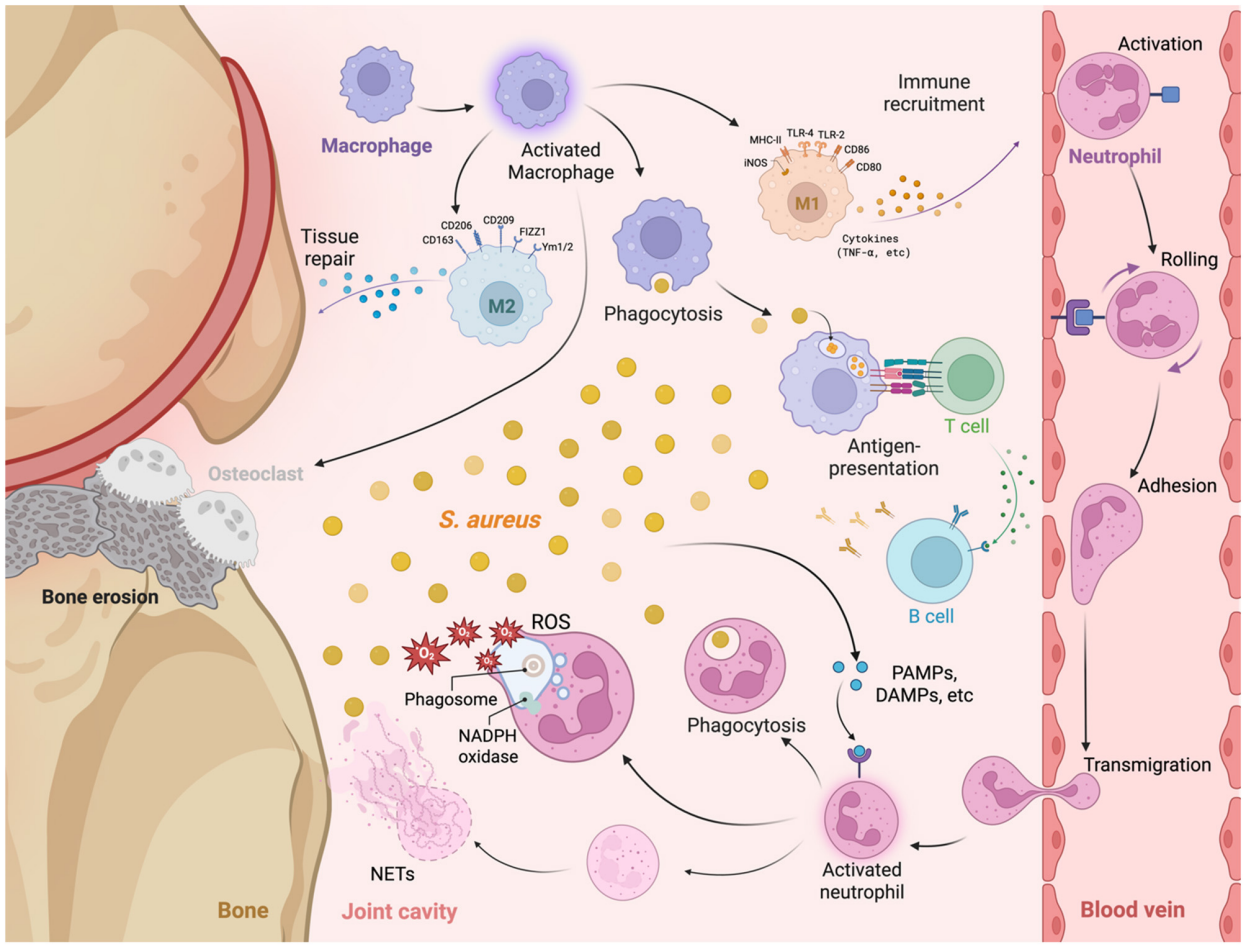
The immune mechanisms activated during *Staphylococcus aureus* septic arthritis, focusing on the interactions between various immune cells and their roles at the site of infection. At the site of infection, *S. aureus* initiates an immune response characterized by the recruitment and activation of macrophages and neutrophils. Upon encountering *S. aureus*, macrophages undergo activation and polarization into distinct phenotypes: M1 Macrophages: Characterized by the expression of markers including MHC-II, TLR4, TLR2, CD80, and inducible nitric oxide synthase (iNOS), M1 macrophages mediate pro-inflammatory responses by secreting cytokines such as TNF-α, thereby facilitating immune cell recruitment and enhancing phagocytosis of *S. aureus*. M2 Macrophages: In contrast, M2 macrophages are involved in anti-inflammatory responses and tissue repair, identified by the expression of markers such as CD206, CD163, CD209, FIZZ1, and Ym1/2. M2 macrophages contribute to the resolution of inflammation and the repair of damaged tissues. Osteoclasts: reabsorb calcium and cause bone erosion. Neutrophils are recruited to the site of infection through a multi-step process involving rolling, adhesion, activation, and transmigration across the endothelial barrier. Upon activation, neutrophils engage in phagocytosis (engulfment and degradation of *S. aureus*). Reactive oxygen species (ROS) production [within phagosomes, nicotinamide adenine dinucleotide phosphate (NADPH) oxidase facilitates the generation of ROS, leading to bactericidal activity]. Neutrophil extracellular traps (NETs) are released by activated neutrophils and are composed of decondensed chromatin and antimicrobial proteins to trap and neutralize extracellular *S. aureus*. Antigen presentation and adaptive immune response: Macrophages and other antigen-presenting cells (APCs) process and present *S. aureus* antigens to T cells, initiating the adaptive immune response and assisting in the activation of B cells. Upon activation, B cells produce specific antibodies against *S. aureus*, which facilitates bacterial clearance and contributes to immunological memory.

Immune response plays a key role in the pathogenesis of *S. aureus* septic arthritis. The host's innate and adaptive immune systems work together to detect and eliminate bacterial infections. However, with the help of its multiple virulence factors, *S. aureus* is able to evade the immune system's attack in various ways, resulting in increased bacterial survival in the host. This section will provide a detailed discussion of how the immune system responds to *S. aureus* infection, including the immune cells and mechanisms involved, with a focus on neutrophils, monocytes, lymphocytes, and TLR2 (Figure 3).

#### 3.2.1 Innate immunity

The innate immune system, which is characterized by its rapid response rather than specificity, serves as the first line of defense when the host vs. pathogen invasion occurs. It identifies PAMPs and damage-associated molecular patterns (DAMPs) to activate a series of immune responses. The innate immune system plays a vital role in septic arthritis caused by *S. aureus*: the complement system, a cascade of plasma proteins, is a crucial effector mechanism for opsonizing pathogens, recruiting immune cells, and directly lysing target cells; innate immune cells, such as neutrophils, monocytes/macrophages and NK cells, limit the bacterial proliferation and spread by phagocytosis, killing and releasing different cytokines. In addition, the innate immune system also recognizes the specific components of *S. aureus* through PRRs, such as TLRs, which activate the downstream signaling pathways and trigger an inflammatory response.

##### 3.2.1.1 Neutrophils

Neutrophils are the most abundant white cells in humans (about 50%−70%) and mice (about 10%−25%) peripheral blood (Hidalgo et al., 2019). Working alongside other resident immune cells, they contribute to the first line of innate immunity to combat *S. aureus* infections. Through chemotaxis, neutrophils swiftly migrate to the site of infection and eliminate pathogens using various mechanisms.

Chemotaxis: Neutrophils can quickly migrate to the infected site by bacterial stimulation and by the compounds *S. aureus* produce or secrete (such as PSMs; and N-formyl-Met-Ile-Phe-Leu, fMIFL) and chemokines secreted by host cells [such as interleukin-8 (IL-8)].

Phagocytosis: Neutrophils can engulf and kill the bacteria through phagocytosis. After *S. aureus* has been encountered and engulfed, the phagosome of neutrophils fuses with the lysosome to form a phagolysosome, which degrades the bacteria through the acidic environment and the action of lysosomal enzymes. This fusion is also important for the oxidative burst, which allows the assembly of the NADPH oxidase complex and the production of ROS both inside the phagolysosome and outside of the cell.

Oxidative burst: Neutrophils produce ROS through the NADPH oxidase system, such as superoxide (${\text{O}}_{2}^{-}$) and hydrogen peroxide (H_2_O_2_), which have a strong bactericidal effect. However, *S. aureus* employs a complicated defense to resist oxidative killing. The bacterium first converts superoxide anions into the less toxic H_2_O_2_ through the action of superoxide dismutase (SodA and SodM). The resulting H_2_O_2_ is then rapidly neutralized into water and oxygen by the crucial enzyme catalase (KatA). Beyond these enzymatic defenses, *S. aureus* utilizes additional strategies, such as the production of carotenoid pigments that quench ROS, and the deployment of repair systems to fix oxidative damage. The collective function of these mechanisms allows *S. aureus* to survive within the neutrophil phagosome. For a comprehensive overview, see (Mandell, 1975; Spaan et al., 2013).

A recent study showed that while PSMα3 strongly activates neutrophil NADPH oxidase via FPR2, PSMβ1 acts as a dual FPR1/FPR2 agonist that partially inhibits PSMα3-induced oxygen radical production, suggesting a protective role against PSMα toxicity (Hu et al., 2022).

NETs: Neutrophils can release extracellular reticule-like structures, which are composed of DNA and antibacterial proteins, which can capture and kill bacteria (Meier et al., 2024). NETs can not only directly kill *S. aureus*, but also limit the spread of bacteria at the infected site (Brinkmann et al., 2004). However, as mentioned in Section 2.2.5, *S. aureus* counteracts this defense by secreting potent nucleases (most notably Nuc) that efficiently degrade the DNA backbone of NETs, allowing it to escape the trap and continue disseminating (Spaan et al., 2013; Flannagan et al., 2015).

##### 3.2.1.2 Monocytes/macrophages

Monocytes, constituting only 5%−10% of the total white cell population in both humans and mice (Seidler et al., 2010), play a considerable role in *S. aureus*-induced septic arthritis. Monocytes circulate in the blood as precursor cells; they can differentiate into macrophages or dendritic cells (DCs) upon recruitment to the site of infection, where they help regulate inflammatory responses. Activated macrophages are not only an outstanding phagocyte but also potent secretors of cytokines and chemokines (Meierovics and Cowley, 2016).

Tissue-resident macrophages are specialized subsets located in specific organs, such as osteoclasts in bone, alveolar macrophages in the lung, microglia in the brain, Langerhans cells in the skin, and Kupffer cells in the liver (Zhao et al., 2024). These cells play highly diverse functions in their tissue localization.

Phagocytosis: Macrophages are capable of recognizing and engulfing *S. aureus*. Following phagocytosis, bacteria are degraded through acidic environments and lysosomal enzymes. Moreover, macrophages can eliminate intercellular pathogens via autophagy, a process involving recruitment of the autophagy protein LC3 to phagosomes, which is induced by signaling through receptors like TLRs and FcγRs (Huang et al., 2009).

Antigen-presentation: In addition to phagocytosis and bacterial killing, macrophages serve as antigen-presenting cells (APCs), processing and presenting bacterial antigens to T cells and activating adaptive immune responses (Banchereau and Steinman, 1998). This process is essential for clearing persistent infections and establishing immune memory.

Inflammation regulation: Macrophages secrete a variety of cytokines, such as TNF-α, IL-6, IL-10, and IL-12, modulating inflammatory response and recruiting additional immune cells (Jin et al., 2021). However, excessive inflammatory response may lead to tissue damage. Macrophages are pathogenic in inducing bone damage in septic arthritis in a murine model, indicating that their activity must be finely regulated (Hu et al., 2025).

Macrophages can polarize into distinct functional types in response to microenvironmental cues. Classically activated (M1) macrophages exhibit potent bactericidal and inflammatory effects, while alternatively activated (M2) macrophages are involved in tissue repair and the resolution of inflammation. The balance between M1 and M2 polarization in response to *S. aureus* infection significantly impacts infection outcomes by modulating immune responses and inflammatory processes (Nguyen et al., 2023; Mohammad et al., 2019, 2022; Hu et al., 2025; Kopparapu et al., 2021; Schultz et al., 2022).

As mentioned earlier, neutrophils are known to be protective during infections, and their depletion leads to worsened outcomes. In contrast. Macrophage depletion attenuates joint swelling and bone damage. Our previous work demonstrated that monocyte depletion in a mouse model of septic arthritis significantly reduced bone resorption induced by *S. aureus* lipoproteins (Schultz et al., 2022). Supporting a protective role for neutrophils and a pathogenic role for monocytes in septic arthritis.

Another study using a *Pseudomonas aeruginosa*-induced septic arthritis mouse model reported similar findings: neutrophils confer a protective role in septic arthritis, whereas monocytes/macrophages are essential for preventing mortality (Jin et al., 2019). These results underscore the dual role of monocytes in both controlling and contributing to the pathology of septic arthritis.

##### 3.2.1.3 Complement system

The host complement system plays a crucial role in the recognition, opsonization, and elimination of invading microbes. This function is carried out by a complex and tightly regulated network of serum proteins and cell surface receptors that act as substrates, enzymes, and modulators within a series of extracellular proteolytic cascades (Walport, 2001a,b).

Complement activation occurs through three well-defined pathways: the classical, lectin, and alternative pathways. All three cascades converge at the central cleavage of C3, producing the active fragments C3a and C3b. The covalent attachment of C3b to foreign surfaces, such as bacterial membranes, promotes opsonization and subsequent phagocytosis by neutrophils and macrophages. Furthermore, C3b amplifies complement activation by forming surface-bound C3 convertases and assembling C5 convertases (Walport, 2001a,b).

Cleavage of C5 initiates formation of the membrane attack complex (MAC), which induces cell lysis. Meanwhile, the anaphylatoxins C3a and C5a are released, triggering potent chemotactic and pro-inflammatory responses that recruit and activate additional phagocytes, thereby enhancing bacterial clearance.

*S. aureus* infections activate all three complement pathways. The classical pathway is typically initiated by antibody-antigen complexes formed between *S. aureus* and host immunoglobulins. However, SpA, which exhibits high affinity for the FC region of IgG, inhibits IgG hexamerization and subsequent C1q recruitment, thereby blocking complement activation on the *S. aureus* surface (Cruz et al., 2021). The lectin pathway is activated by mannose-binding lectin (MBL) binding to *S. aureus* surface components, such as wall teichoic acid (Park et al., 2010; Gerlach et al., 2018). The alternative pathway is activated on the bacterial surface and is sustained through an amplification loop involving factor B, factor D, and properdin (Harboe and Mollnes, 2008).

The importance of complement in septic arthritis has been demonstrated in multiple experimental models. Complement depletion using cobra venom factor in murine septic arthritis resulted in significantly more severe clinical arthritis and bone destruction (Sakiniene et al., 1999). Pathway-specific studies further reveal distinct roles: in cecal ligation and puncture–induced sepsis, factor D-deficient mice (impaired in alternative pathway activation) retained adequate bacterial clearance, whereas C1q-deficient mice (lacking classical pathway activation) demonstrated compromised bacterial clearance (Dahlke et al., 2011). Similarly, the lectin pathway has been shown to be essential for antimicrobial defense in this model (Windbichler et al., 2004). In a murine model of *S. aureus* septic arthritis, C3-deficient mice develop more severe disease with higher bacterial burdens in the kidneys, whereas factor B-deficient mice do not exhibit this phenotype (Na et al., 2016). These findings demonstrate the essential role of central complement activation, while suggesting a more limited contribution of the alternative pathway in host defense against septic arthritis and related infections.

#### 3.2.2 Adaptive immunity

The adaptive immune system serves as the second line of defense. It is characterized by high specificity and strong memory, providing long-lasting protection through the production of antibodies and effector T cells after the first exposure to pathogens. In septic arthritis caused by *S. aureus*, the role of the adaptive immune system is crucial. *S. aureus* can not only trigger a strong initial immune response, but also evade immune surveillance through a variety of mechanisms, leading to reinfections (Kwon et al., 2022; Corrado et al., 2016).

##### 3.2.2.1 T cells

CD4+ T helper cells (Th cells) regulate the function of other immune cells by secreting cytokines. They play an important role in *S. aureus* infection, especially the Th1 and Th17 cell subgroups, as reviewed in Dong (2021). Th1 cells promote the activation and bactericidal effect of macrophages by secreting IFN-γ, while Th17 cells promote the collection and activation of neutrophils by secreting interleukin-17 (IL-17; McGeachy et al., 2019).

Regulatory T cells (Tregs) are primarily CD4+ and play a crucial role in maintaining immune tolerance during *S. aureus* infection, thereby preventing excessive inflammation and tissue damage. However, their impact on pathogen clearance requires careful consideration for effective management of chronic infections and reinfections (Sakaguchi et al., 2009; Belkaid and Rouse, 2005).

CD8+ cytotoxic T cells, although essential for combating intracellular pathogen infection, have not been demonstrated to serve a significant role in *S. aureus* infections. CD8+ T cells typically kill infected cells and limit bacterial spread by releasing perforin and granzymes. For a comprehensive overview, see Harty et al. (2000). In addition, CD8+ T cells can secrete a variety of cytokines, such as IFN-γ, TNF-α, IL-2, to regulate the balance of the immune response (Gerlach et al., 2013).

Various *in vivo* studies related to *S. aureus*-induced septic arthritis have demonstrated that T cells serve different roles depending on the T cell subtype involved, the modulation of T cell activity, or the effects of subjecting mice to certain purified *S. aureus* components upon induction of septic arthritis. Interestingly, depletion of CD4+ T cells diminished the arthritogenic outcome, hence indicating that CD4+ T cells provoke septic arthritis severity, whereas depletion of CD8+ T cells displayed a tangible effect on the severity of the disease (Abdelnour et al., 1994b). Conversely, a previous study demonstrated that inhibition of the T cell activity via the immunosuppressive agent, CTLA4-Ig, promotes the disease progression (Ali et al., 2015a). However, the role of T cells in our mouse model, while challenged with purified *S. aureus* Lpp intra-articularly into the knee joint, was found to be of less importance, as double depletion of CD4+ and CD8+ T cells, or CTLA4-Ig therapy, displayed similar effects as their respective controls (Mohammad et al., 2019).

##### 3.2.2.2 B cells and antibodies

B cells produce antibodies that target specific surface antigens on pathogens and differentiate into plasma cells, a process that can be further enhanced by T helper cells. These antibodies can neutralize bacterial toxins, prevent bacteria from adhering and invading host cells, and remove bacteria by activating the complement system and promoting phagocytosis.

B cells play a crucial role in the immune response against *S. aureus* infection by producing specific antibodies, primarily IgM, IgG, and IgA. IgM is the first antibody produced in response to infection, offering an initial defense (Gupta et al., 2023). IgG provides systemic protection throughout the serum, while IgA is crucial for local immunity on the surface of the mucosa (Abbas et al., 2020). After the initial infection, some B cells differentiate into memory B cells, when encountering the same pathogen again, they can quickly proliferate and produce a large number of high-affinity antibodies, providing a faster and more effective immune response (Abbas et al., 2020). However, *S. aureus* has a variety of strategies to avoid antibody-mediated immunity, such as binding to the antibody Fc segments via SpA and *S. aureus* binder of immunoglobulin (Sbi) to prevent the neutralization and phagocytosis function of antibodies, as well as producing extracellular polysaccharides and biofilms to protect itself from the attack of antibodies and the complement system (Foster, 2005; Otto, 2013; Zecconi and Scali, 2013).

B cells are considered less potent in involving the pathogenesis of *S. aureus* septic arthritis. Previous studies have shown that the depletion of B cells in mouse models did not contribute to the development of arthritis, mortality, or bacterial clearance (Gjertsson et al., 2000). However, these experimental findings derive exclusively from juvenile, immunocompetent murine models raised under specific pathogen-free (SPF) conditions. These animals exhibit antigen-inexperienced B cell compartments due to the absence of prior *S. aureus* exposure, which likely underlies the observed attenuation of B cell-mediated protective efficacy. Future studies should define B cell functionality in antigen-primed models featuring controlled pathogen reexposure or adoptive transfer of immune memory.

#### 3.2.3 Receptors

**Figure 4:**
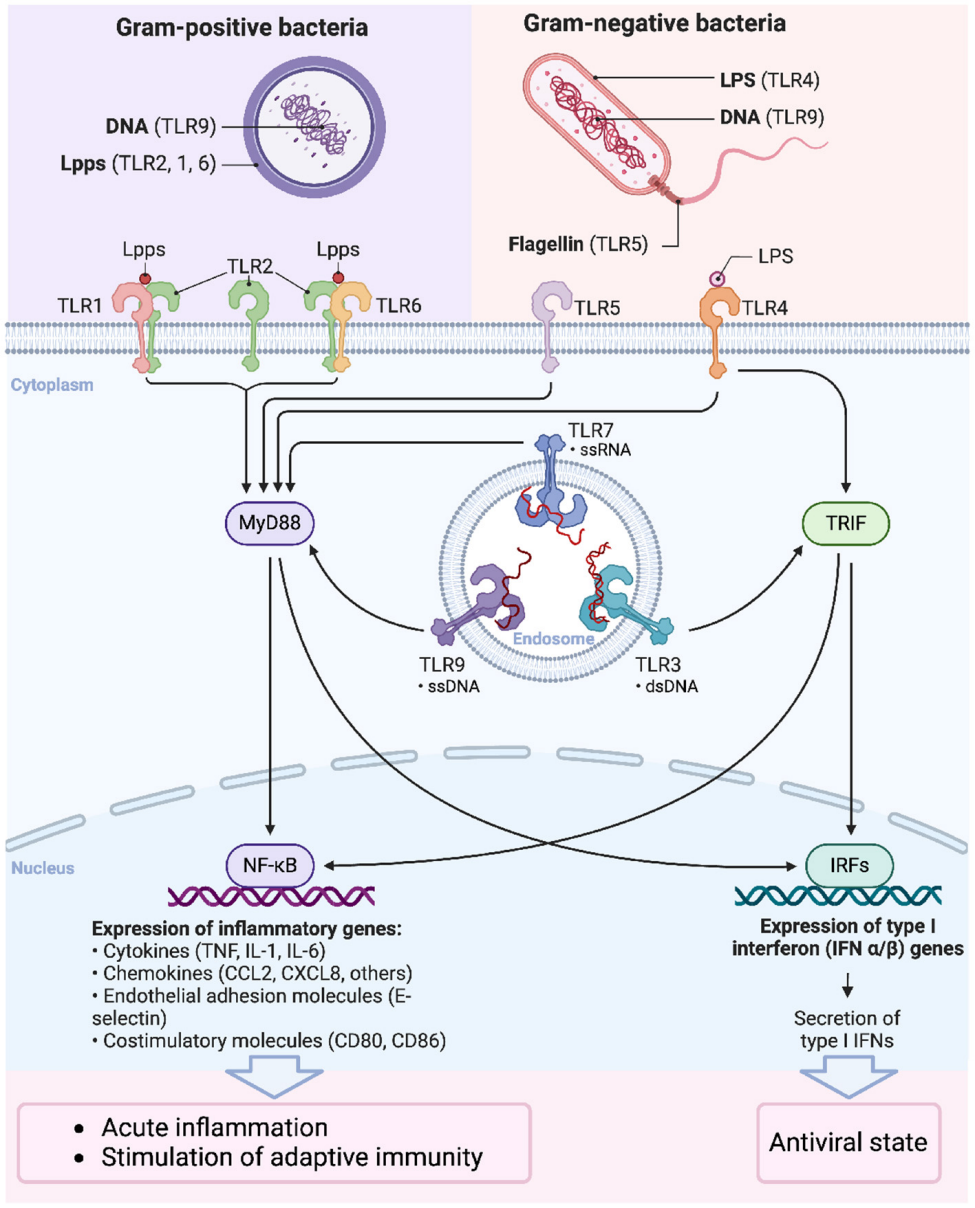
Signaling functions of Toll-like receptors. The signaling pathways activated by Toll-like receptors (TLRs) upon recognition of pathogen-associated molecular patterns (PAMPs) from Gram-positive and Gram-negative bacteria. TLRs are critical components of the innate immune system, recognizing distinct microbial components and initiating downstream signaling that leads to immune responses. Bacterial DNA from Gram-positive bacteria is recognized by TLR9. Lipoproteins (Lpps) are the predominant agonists of TLR2. Upon Lpp recognition, TLR2 forms heterodimers with TLR1 or TLR6, depending on the lipid portion structure of the Lpp. Lipopolysaccharide (LPS) from Gram-negative bacteria is recognized by TLR4, bacterial DNA by TLR9, and flagellin by TLR5. MyD88-dependent pathway: Most TLRs, including TLR1, TLR2, TLR4, TLR5, TLR7, and TLR9, signal through the adaptor protein MyD88, leading to the activation of NF-κB. This transcription factor regulates the expression of pro-inflammatory cytokines (e.g., TNF-α, IL-1, IL-6), chemokines, adhesion molecules, and costimulatory molecules, resulting in acute inflammation and the stimulation of adaptive immunity. TRIF-dependent pathway: TLR3 and TLR4 can signal through the adaptor protein TRIF, leading to the activation of interferon regulatory factors (IRFs), which promote the expression of type I interferons (IFN-α/β). This pathway establishes an antiviral state within the host cells.

Receptors trigger a series of immune responses and signaling pathways by recognizing PAMPs and DAMPs. In addition to the widely studied TLRs (Figure 4), other receptors, such as FPR and the receptor for Advanced Glycation Endproducts (RAGE), involved in *S. aureus* infection have also been gradually revealed. FPR is an important receptor for the recognition of bacterial formyl peptides and participates in the chemotaxis and activation of neutrophils, while RAGE plays a significant role in inflammatory responses and tissue damage (Mohammad et al., 2016). In this section, the specific functions of TLR2 and FPR receptors will be discussed in depth in regard to *S. aureus* septic arthritis and their latest research progress, elucidating their key roles in pathogen recognition and host defense.

##### 3.2.3.1 TLRs

TLRs were the first PRRs identified in mammals, as more than 10 TLRs have been discovered so far, most of them being capable of recognizing different PAMPs (El-Zayat et al., 2019). Particularly, TLR2 has an essential role in detecting diverse PAMPs from bacteria, particularly lipid-based bacterial cell wall components like lipoproteins. Upon detection, TLR2 activates a series of downstream signaling pathways that elicit the host's immune response (Takeuchi and Akira, 2010).

TLR2 is a transmembrane protein; the extracellular region of TLR2 contains multiple leucine-rich repeats (LRRs) that are responsible for recognizing PAMPs. The intracellular region of TLR2 contains a TIR (Toll-IL-1 receptor) domain that is responsible for interacting with downstream signaling molecules (O'Neill et al., 2013). TLR2 usually forms heterodimers with TLR1 or TLR6 to enhance its ability to recognize different PAMPs. For example, TLR2/TLR1 heterodimers primarily recognize triacylated lipoproteins, while TLR2/TLR6 heterodimers recognize diacylated lipoproteins (O'Neill et al., 2013).

After binding to its ligands, TLR2 gets activated and recruits myeloid differentiation factor 88 (MyD88) and TIR domain-containing moderate protein (TIRAP) through the intracellular TIR domain, initiating downstream signaling pathways (Kawai and Akira, 2010). These signaling pathways include NF-κB and MAPK pathways, which ultimately lead to the production and release of inflammatory mediators such as TNF-α, interleukin-12 (IL-12), and interleukin-6 (IL-6; Akira, 2009).

In septic arthritis, TLR2 was found to play a pro-inflammatory and catabolic role mediated by the NF-κB pathway (Papathanasiou et al., 2011). Mice lacking TLR2 showed reduced frequency and less severe arthritis compared to controls in an antibiotic-killed *S. aureus*-induced arthritis model (Ali et al., 2015b). Another study demonstrated that the regulation of IgG relied on TLR2, but not IgM, in the humoral immune response to infection (Gupta et al., 2023). In addition, TLR2-mediated signaling pathways also play an important role in promoting the chemotaxis and activation of neutrophils and monocytes/macrophages (Kurt-Jones et al., 2002). Both TLR2 deficiency and aging were found to compromise immune response. *In vitro*, the absence of TLR2 and advanced age led to reduced cytokine and chemokine production by peritoneal macrophages and splenocytes. *In vivo*, the disease severity mainly depended on the presence of TLR2. Both young mice and old mice lacking TLR2 experienced more significant weight loss, in contrast to their WT counterparts. Notably, aged TLR2-deficient mice exhibited the most severe weight loss and harbored the highest bacterial load in the kidney among all groups (Hu et al., 2023). This paradox, wherein TLR2 deficiency protects against joint-specific inflammation but exacerbates systemic bacterial dissemination, highlights the complex dual role of TLR2 and presents a significant challenge for therapeutic targeting. It suggests that global inhibition of TLR2, while potentially beneficial for controlling local inflammatory damage, might be harmful by compromising systemic host defense during bacteremia.

In clinical studies, polymorphisms of TLR2 are closely related to the susceptibility and severity of various infectious diseases. Certain mutations in the TLR2 gene may increase the host's susceptibility to *S. aureus* infections, which provides a potential target for personalized treatment (Schroder and Schumann, 2005). In addition, immunomodulators and anti-inflammatory drugs targeting the TLR2 signaling pathway are also being studied, showing a promising role in the treatment of *S. aureus* infection and related diseases (Caplan and Maguire-Zeiss, 2018). Future therapeutic strategies should therefore be carefully considered, potentially favoring localized modulation (e.g., intra-articular application) over systemic inhibition to avoid adverse outcomes.

##### 3.2.3.2 FPRs

**Figure 5:**
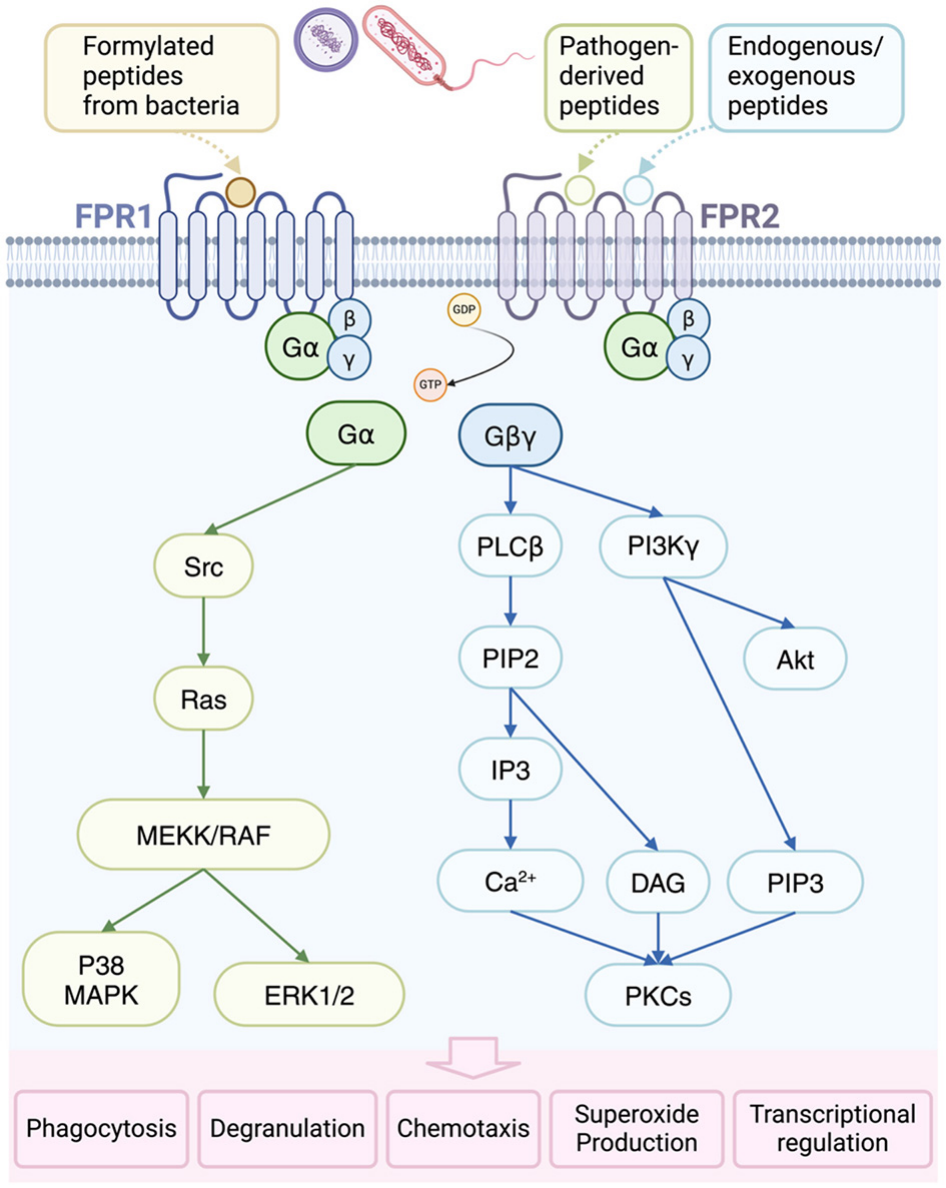
Activation and consequences of formyl-peptide receptor (FPR) in bacterial infections. FPR1 is primarily activated by formylated peptides from bacteria and mitochondria, while FPR2 responds to specific endogenous and pathogen-derived ligands. Upon activation, FPRs facilitate the conversion of guanosine diphosphate (GDP) to guanosine triphosphate (GTP), leading to the dissociation of heterotrimeric G proteins into α and βγ subunits. The βγ subunit activates phospholipase Cβ (PLCβ), resulting in the release of calcium from intracellular stores and subsequent activation of protein kinases C (PKCs). Additionally, the βγ subunit triggers phosphoinositide-3-kinase γ (PI3Kγ), further promoting PKC and protein kinase B (Akt) activation. The α subunit, on the other hand, activates Ras superfamily GTPases, contributing to the activation of the MAPK pathways, p38, and extracellular-regulated protein kinase 1/2 (ERK1/2). These signaling cascades ultimately lead to various cellular responses, including phagocytosis, degranulation, chemotaxis, superoxide anion production, and transcriptional activation. DAG, diacylglycerol; MEKK, MAP kinase kinase; RAF, rapidly accelerated fibrosarcoma; PIP, phosphatidylinositol 4,5-bisphosphate; IP3, inositol 1,4,5-trisphosphate.

Formyl Peptide Receptors (FPRs) belong to the G protein-coupling receptors (GPCRs) family, which are mainly responsible for recognizing and responding to formyl peptides and other related molecules produced by bacteria. Three human FPRs are found on the surface of various cells. Both monocytes and neutrophils express FPR1 and FPR2, whereas FPR3 is expressed in monocytes but absent in neutrophils. FPR1 and FPR2 share 69% amino acid sequence identity and signal through similar pathways (Weiss and Kretschmer, 2018). In *S. aureus* infection, FPR1 and FPR2 are the two prominent FPR members, which are known to enhance the host's innate immunity for eliminating pathogens, by mediating chemotaxis and activation of neutrophils and other immune cells (Figure 5; Sundqvist et al., 2019; Lebtig et al., 2023).

The mouse FPR gene family comprises eight identified members, while most of them are expressed in neutrophils, research has mainly focused on *mFpr1* and *mFpr2*. These genes are extensively expressed in mouse phagocytic leukocytes and exhibit a high degree of homology to their human analogs (He and Ye, 2017).

FPR1 was originally discovered due to its high affinity for bacterial formyl peptides such as N-formyl-Met-Leu-Phe (fMLF). FPR1 is mainly expressed on the surface of innate immune cells such as neutrophils and monocytes (Le et al., 2002). When FPR1 recognizes and binds formyl peptides such as fMLF, it initiates a series of downstream signaling pathways, including the phosphatidylinositol-3-kinase (PI3K) pathway and the MAPK pathway, which leads to intracellular calcium mobilization and protein kinase C (PKC) activation (Snapkov et al., 2016; Gemperle et al., 2012). These changes ultimately result in chemotaxis, degranulation, and ROS production from neutrophils, which enhance their ability to eliminate pathogens (Gemperle et al., 2012; Dahlgren and Karlsson, 1999).

FPR2, initially considered to be a close relative of FPR1, has been found to not only recognize fMLF (low affinity), but also distinguish a variety of endogenous and exogenous ligands, such as lipoxin A4 and serum amyloid A (SAA; Bena et al., 2012; Tylek et al., 2021). The ligand diversity of FPR2 gives it a wider range of functions, including the dual regulation of anti-inflammatory and pro-inflammatory effects (He and Ye, 2017; Ye et al., 2009). In neutrophils and macrophages, FPR2 regulates the chemotaxis and cell activation through signaling pathways similar to FPR1, but its response to anti-inflammatory signaling molecules plays a unique role in regulating the inflammatory response, which may open the door for developing new therapeutic approaches (Dufton and Perretti, 2010; Hardesty et al., 2023).

FPR1 and FPR2 not only play a role in initiating and maintaining the inflammatory response, but also participate in the immune regulation processes. Especially by combining anti-inflammatory molecules, such as lipoxin A4, excessive inflammatory reactions can be inhibited, thereby preventing tissue damage (Migeotte et al., 2006). In *S. aureus*-induced infections, the activation of FPR1 and FPR2 increases the migration of neutrophils to infection sites and enhances bacterial phagocytosis. Specifically, PSMs released by *S. aureus* activate these receptors, resulting in enhanced phagocytosis and bacterial killing by neutrophils (Weiß et al., 2020). Indeed, as shown before (Hu et al., 2022), neutrophils are activated by PSMα via FPR2, whereas PSMβ, a conditional weak agonist, can activate TNF-α-primed neutrophils through both FPR1 and FPR2. This indicates the significant role these two receptors play in recognizing and eliminating pathogens.

### 3.3 Sex and hormonal changes

Sex and hormonal changes are considered as one of the important factors in the host's susceptibility and disease progression. sex differences and sex hormone levels have a significant impact on the regulation of immune responses, thereby affecting the severity and outcomes of *S. aureus* infections.

The incidence and mortality rate of males in a variety of infectious diseases are usually higher than those of females (Dhakal et al., 2022). This may be related to some characteristics of the male immune system. For example, healthy males usually show higher levels of inflammatory cytokines, such as TNF-α and IL-6, as reviewed in Klein and Flanagan (2016). Although these cytokines help to quickly eliminate pathogens, they may also trigger excessive inflammatory responses and lead to tissue damage (Chen et al., 2018; Moller and Villiger, 2006).

In contrast, females tend to have a stronger immune response to infections. This is closely related to the immunomodulatory effects of estrogen. Estrogen can enhance the function of B cells and T cells, improve antibody production and cell-mediated immune responses, as well as upregulation autoantibodies (Verthelyi, 2001). However, an excessive immune response may also increase the risk of autoimmune diseases in females (Verthelyi, 2001; Bouman et al., 2005). For example, RA and systemic lupus erythematosus (SLE), the diseases that are much more prevalent in females, are in turn known risk factors for septic arthritis.

Several studies have indicated that the immunomodulatory effects of sex hormones and gender-based differences in X chromosome gene expression might significantly impact treatment outcomes in various diseases (Dias et al., 2022; Grimaldi et al., 2005; Prajapati et al., 2022). Multiple nationwide and hospital-based epidemiological studies have reported a higher prevalence of sepsis in men compared to women (Lakbar et al., 2023). However, clinical study findings have been inconsistent. For example, women with septic arthritis tend to be older and have more pre-existing joint conditions than men, though there were no major differences in clinical presentation, treatment, or outcomes (Nissim et al., 2021). Another retrospective study, including 1,348 patients, showed that no significant difference was found between male and female patients with sepsis (Eachempati et al., 1999). In a murine model of *S. aureus* bacteremia, sex had no major impact on mortality, kidney abscesses, or bacterial loads (Hu et al., 2023; Gupta et al., 2023). These discrepancies in study results may be due to variations in sex steroid levels among patients rather than the specific types of sex hormones alone.

### 3.4 Genetic susceptibilities

Septic arthritis caused by *S. aureus* exhibits significant variation among individuals in susceptibility and disease progression. These differences are not only influenced by environmental factors and immune status but are also closely related to the genetic background of the host. In recent years, genetic studies have revealed the role of multiple genes involved in immune system function and inflammatory response in septic arthritis.

Through genome-wide association analysis (GWAS) and candidate gene studies, several studies have revealed the relationship between key genes and polymorphisms associated with *S. aureus* infections. For example, a GWAS study found that TLRs (especially TLR2) and NOD2 gene polymorphisms are associated with high *S. aureus* infection susceptibility (Kanneganti et al., 2007; Stappers et al., 2014; Lorenz et al., 2000). A study involving 155 patients with infections and 262 healthy controls demonstrated that variants in TLR2 (rs5743708) and TLR4 (rs4986790) are linked to an increased susceptibility to severe infections. Specifically, the TLR2 polymorphism was associated with a 3.16-fold higher risk of recurrent infections (Teräsjärvi et al., 2024). Another study identified a novel polymorphism in the TLR2 gene (Arg753Gln) that may increase susceptibility to staphylococcal infections and septic shock (Lorenz et al., 2000). In addition, specific polymorphisms in the IL-1β and TNF-α genes have been shown to correlate with disease severity and prognosis (El-Tahan et al., 2016; Dinarello, 2011).

Interestingly, the expression level of the *S100a8/a9* gene may predict *S. aureus*-induced septic arthritis in a mouse model (Deshmukh et al., 2023), indicating the possibility of using it as a potential biomarker to forecast the evolution of disease and seek more effective therapeutic strategies.

### 3.5 Osteoimmunology and bone damage in septic arthritis

As mentioned above, the bone destruction seen in septic arthritis is permanent. Bone undergoes continuous remodeling throughout life, involving a delicate balance between resorption and formation, primarily driven by two distinct cell types. Osteoclasts are responsible for breaking down and resorbing bone calcium, while osteoblasts contribute to bone formation by depositing calcium.

In the pathological process of septic arthritis, bone tissue plays an active role in the immune response rather than being merely a passive victim. The immune response in the skeletal system involves complex interactions between immune cells and bone cells, and the concept of “osteoimmunology” was initially pointed out to describe the interface between them in 2000 (Arron and Choi, 2000).

In a mouse model of *S. aureus* osteomyelitis, immune cells are mobilized and attracted to the infection site by elevated levels of potent neutrophil-attracting chemokines produced by osteoblasts in the bone tissue. These chemokines, including CXCL1, CXCL2, CXCL3, CXCL5, CCL3, and CCL7, promote osteoclastogenesis and leukocyte recruitment, driving the inflammatory response to bacterial invasion (Sipprell et al., 2023). It has been shown that bone marrow macrophages and osteoclasts play crucial roles in the inflammatory response by releasing inflammatory factors and mediators, which initiate osteoclastogenesis, recruit leukocytes, and result in bone remodeling (Torres et al., 2023).

**Figure 6:**
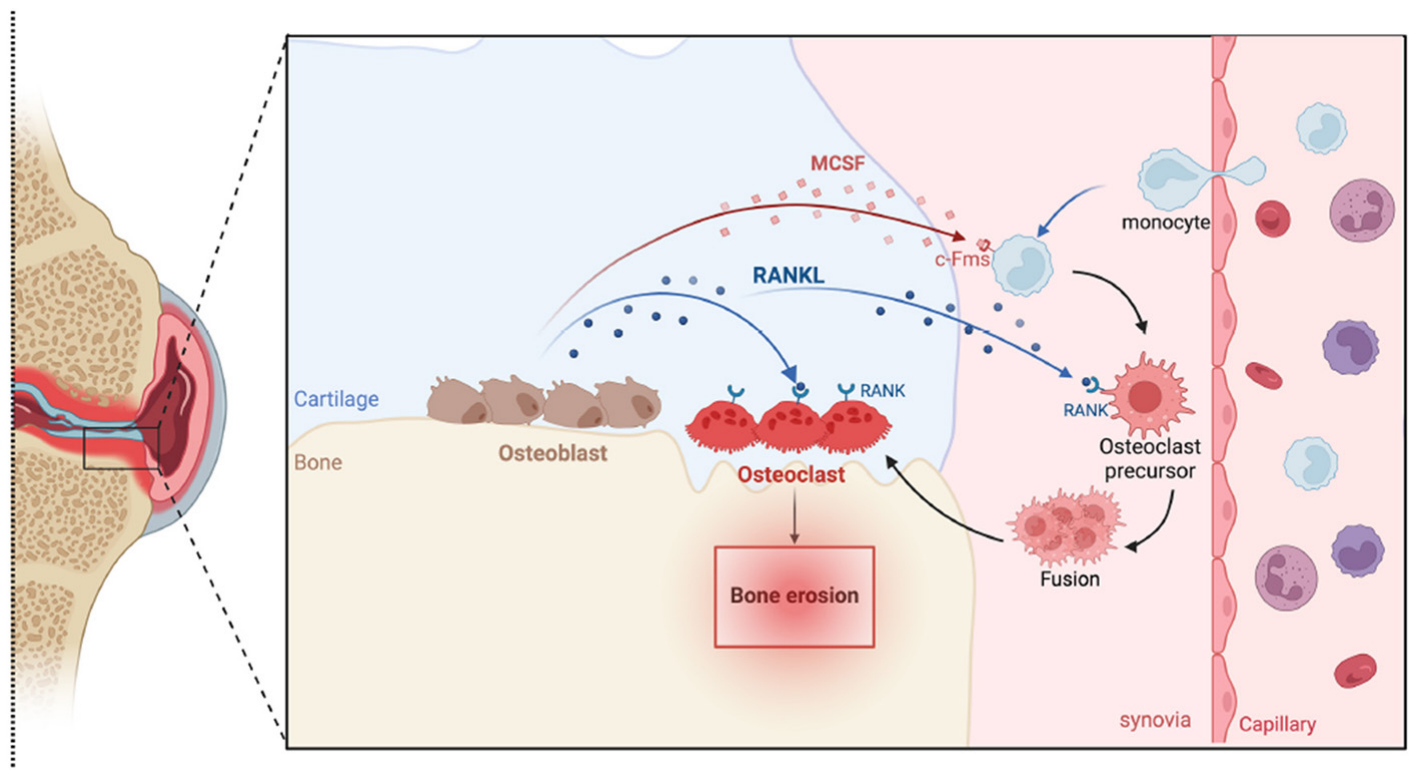
Schematic illustration of bone osteoclastogenesis. Monocytes from capillaries differentiate into osteoclast precursors in response to macrophage colony-stimulating factor (M-CSF) and receptor activator of nuclear factor kappa-*B* ligand (RANKL). These precursors express receptors such as c-fms for M-CSF and RANK for RANKL. When these receptors bind to their respective ligands, produced by osteoblasts, they trigger intracellular signaling cascades that promote the fusion of these precursors into mature, multinucleated osteoclasts, which are capable of bone resorption.

A series of cytokines and chemokines tightly regulate bone remodeling. For instance, the RANKL, also known as OPGL or TRANCE, was identified in 1999 and is expressed on the surface of different cells, especially on osteoblasts and activated T cells (Wong et al., 1999). RANKL, in conjunction with macrophage-colony stimulating factor (M-CSF), plays a key role in regulating osteoclastogenesis (Kwan Tat et al., 2004). M-CSF binds to its receptor, c-Fms, which triggers the differentiation of hematopoietic stem cells (HSCs) into the monocyte/macrophage lineage and promotes the survival and proliferation of preosteoclast (Fuller et al., 1993; Xaus et al., 2001). Subsequently, RANKL interacts with its receptor activator of NF-κB (RANK) on osteoclast precursors facilitating their differentiation, fusion, and maturation into active osteoclasts (Chandrabalan et al., 2024; Honma et al., 2021). The bone osteoclastogenesis is illustrated in Figure 6.

Osteoclast activity is modulated by several factors, including the increased expression of RANKL, which is influenced by pro-inflammatory cytokines such as TNF-α, IL-1, and IL-6. Osteoclast precursors release these cytokines and can also be stimulated by LPS, further promoting osteoclastogenesis and bone resorption (Kwan Tat et al., 2004; Chaiwut and Kasinrerk, 2022). In conditions like septic arthritis, where *S. aureus* infection triggers intense inflammation, there is a significant increase in osteoclast production and activation, leading to excessive bone resorption and loss of bone mass in the affected joints. Lpps-induced bone resorption has been shown in a mouse model of *S. aureus* local knee infection from our previous studies (Hu et al., 2025; Schultz et al., 2022).

In contrast, OPG, serving as an antagonist of RANKL, inhibits its interaction with RANK by competitively binding to RANKL, thereby blocking the production of osteoclasts (Kondegowda et al., 2023). In addition, denosumab, an osteoporosis medication approved by the U.S Food and Drug Administration (FDA) and the European Medicines Agency (EMA), is a human monoclonal antibody that targets RANKL, inhibiting RANKL/RANK interaction and consequently disrupting osteoclast formation. In the inflammatory milieu, such as that induced by *S. aureus*, the expression of RANKL is increased, while the expression of OPG is reduced, thus promoting bone resorption (Kong et al., 1999). This reduction of OPG promotes osteoclastogenesis and bone destruction in response to *S. aureus* infections. Indeed, it has been shown that blocking RANKL efficiently prevents systemic bone resorption in a mouse model of *S. aureus* septic arthritis (Verdrengh et al., 2010). Remarkably, combination therapy resulted in the lowest bone erosion scores among all groups, which were significantly lower than in mice treated with cloxacillin alone. Furthermore, bone erosion was prevented in the combination group, compared to 20% in the group receiving antibiotics alone.

### 3.6 Bone tissue changes in septic arthritis

Various conditions and diseases can lead to joint disorders. Degenerative joint diseases like osteoarthritis (OA) and inflammatory conditions such as RA exhibit distinct patterns of joint destruction. OA is characterized by new bone formation at its late stage, while RA involves bone resorption (Terashima et al., 2024; Li et al., 2023). In *S. aureus* septic arthritis, bone tissue undergoes significant pathological changes, which include not only bone destruction, but also remodeling of bone structure and alterations in bone density.

Various factors, including aging, health conditions, nutritional status, and inflammatory responses, influence changes in the bone microenvironment. These factors are crucial for maintaining the balance between osteoclasts and osteoblasts, which is essential for healthy bone homeostasis (Choi et al., 2024; Mi et al., 2024). In inflammatory conditions, the imbalance between osteoclasts and osteoblasts potentially leads to osteoporosis and structural alterations in bone composition. Prolonged joint inflammation can result in trabecular thinning, reduced cortical bone thickness, and a significant decrease in bone strength (Schett and David, 2010).

A significant decrease in systemic bone mineral density (BMD) has been reported in patients with RA (Lodder et al., 2004; Sivaprasad et al., 2023). This is due to a chronic inflammatory response, mainly characterized by elevated proinflammatory cytokines, which lead to increased bone resorption and decreased bone formation. The decrease in bone density not only increases the risk of fractures but also further exacerbates joint dysfunction. Additionally, studies on mouse models induced by *S. aureus* and Lpp have further supported these findings, showing a loss of BMD in local knee joints (Schultz et al., 2022; Fatima et al., 2017). Furthermore, the roles of aging and TLR2 in the outcome of BMD levels were also explored. This study also indicated that compared to those TLR2-deficient mice, young mice with the expression of TLR2 showed a BMD reduction after *S. aureus* infection, underscoring the impact of decreased bone density on joint integrity and overall musculoskeletal health (Schultz et al., 2024).

## 4 Therapeutic Implications

The management of septic arthritis has seen limited advancements over the past two to three decades, relying on a combination of antibiotics, and surgical interventions such as joint drainage or debridement. Despite these standard approaches, the prognosis remains suboptimal, with a markedly elevated long-term risk of subsequent prosthetic surgery (Abram et al., 2020). It is therefore essential to better characterize patient profiles, evaluate the impact of bacterial antimicrobial resistance on therapeutic efficacy, and consider the necessity of joint reoperations and the frequency of debridement within current treatments (Hodea et al., 2024). Moreover, enhancing early detection, ensuring timely intervention, adopting multidisciplinary approaches, and implementing antimicrobial therapies are pivotal to developing improved therapeutic strategies (Deshmukh et al., 2023; Berinson et al., 2023; Kim et al., 2023).

Transcriptome sequencing analysis highlights the potential of S100a8/a9 gene expression as a biomarker for predicting septic arthritis development in mice with *S. aureus* bacteremia, aiding in the development of more efficient treatment strategies (Deshmukh et al., 2023). Molecular diagnostic tools like the BioFire joint infection assay (BJA), a PCR-based rapid diagnostic technique approved by the FDA, have shown high sensitivity and specificity in identifying bacterial pathogens, enabling faster species identification and optimization of antibiotic therapy, particularly in cases involving difficult-to-culture bacteria or prior antibiotic treatment (Berinson et al., 2023). Especially, the infection of MRSA strains in native joint septic arthritis poses a higher risk of treatment failure, emphasizing the need for vigilant monitoring and targeted interventions (Kim et al., 2023).

Understanding the intricate interplay between bacterial and host factors is essential for developing innovative therapeutic approaches for septic arthritis. This complex interaction not only influences the disease's progression and severity but also offers specific targets for therapeutic intervention. Therefore, effective treatment approaches must address both bacterial and host factors at multiple levels to mitigate the disease's impact.

### 4.1 Targeting virulence factors treatment

The pathogenicity of *S. aureus* is largely driven by its diverse array of virulence factors, including adhesion molecules, extracellular enzymes, and toxins. Targeting these components represents promising therapeutic approaches for septic arthritis. Vaccination strategies targeting specific virulence factors have shown efficacy across multiple preclinical models, including mice, rabbits, and non-human primates. Although studies focusing on septic arthritis remain limited, both active and passive immunizations against ClfA have been shown to protect against septic arthritis and reduce sepsis-induced mortality (Josefsson et al., 2001). Similarly, vaccination with a recombinant fragment of the *S. aureus* collagen adhesin effectively prevents lethal sepsis (Nilsson et al., 1998).

Despite these promising findings, all human clinical trials of *S. aureus* vaccines have thus failed to prevent invasive infections (Proctor, 2012; Miller et al., 2020). One explanation for this discrepancy is that, unlike animal models, which are often immunologically naïve, humans are frequently colonized by *S. aureus* from early life. This prior exposure may induce an anti-inflammatory response mediated by IL-10, which alters the sialylation of anti-*S. aureus* antibodies and diminishes their protective efficacy (Tsai et al., 2024). Consequently, the non-protective immune imprint generated by prior *S. aureus* exposure may be recalled upon vaccination, such as with the iron-scavenging protein (IsdB), leading to competition between non-protective humoral responses and newly induced protective antibody responses, thereby compromising vaccine effectiveness (Tsai et al., 2022; Caldera et al., 2024). Future efforts are therefore directed toward developing multi-antigen vaccines, disrupting IL-10-mediated immunosuppression, and shifting focus from cell-wall antigens to key toxins (Tsai et al., 2024; Caldera et al., 2024).

In septic arthritis, the surface adhesion molecules ClfA and vWbp of *S. aureus* facilitate the bacterium's adherence to the synovial membrane and cartilage surfaces of the joints, thereby promoting colonization and infection. Targeting these interactions with small-molecule inhibitors, which disrupt the binding between bacterial adhesins and host extracellular matrix components, represents a promising strategy for inhibiting intra-articular colonization and mitigating infection severity (Na et al., 2020; Claes et al., 2017). In addition, certain *S. aureus* toxins, including α-toxin and PSMs, contribute to joint tissue destruction and trigger inflammatory responses. Inhibitors targeting α-toxin have demonstrated protection against tissue damage in murine models of septic arthritis (Nilsson et al., 1999; Wright and Nair, 2010; Gemmell et al., 1997).

Studies further indicate that biofilm formation represents a key mechanism enabling *S. aureus* persistence within joints, enhancing resistance to both host immunity and antibiotic therapy. Drugs that disrupt biofilm integrity or inhibit its formation may improve antibiotic efficacy and reduce rates of recurrence (Otto, 2013; Kwiecinski et al., 2019).

### 4.2 Enhancing host immune responses

The host immune response plays a crucial role in controlling *S. aureus* infection; however, an excessive inflammatory reaction can lead to joint damage. Thus, enhancing joint-specific immune responses while avoiding excessive inflammation is another promising therapeutic strategy for septic arthritis.

TLRs, particularly TLR2, are essential for recognizing *S. aureus* via Lpps and initiating downstream immune signaling. Modulating TLR2 activity offers a means to attenuate inflammation-induced tissue damage while preserving antimicrobial immunity (Takeuchi and Akira, 2010). For instance, certain TLR2 agonists or inhibitors have demonstrated the potential to modulate inflammatory responses through STAT3/SOCS3 signaling in a murine model of septic arthritis (Ghosh and Bishayi, 2024).

Additionally, studies indicate that targeted modulation of intra-articular cytokine networks, such as inhibition of pro-inflammatory cytokines (TNF-α, IL-1β, etc.; Ghosh and Bishayi, 2024; van den Berg, 2001) and metalloproteinases (Gjertsson et al., 2005), significantly mitigates inflammation-driven bone destruction. Research studies on other cytokines, including IL-4 (Hultgren et al., 1998), IL-10 (Gjertsson et al., 2002; Puliti et al., 2002), IL-15 (Henningsson et al., 2012), and IL-33 (Staurengo-Ferrari et al., 2018), have also yielded promising immunomodulatory effects. Specific interventions in bone-remodeling pathways, such as using OPG to counteract the RANKL/RANK axis and inhibit osteoclast activation, have achieved significant success in preventing bone resorption (Verdrengh et al., 2010). More recently, anti-RANKL treatment has been shown to markedly reduce bone erosion in a mouse model of septic arthritis (Hu et al., 2025). Such immunomodulatory approaches, when combined with antibiotics, may improve infection control and preserve joint integrity. Nevertheless, randomized clinical trials are warranted to evaluate their efficacy in preventing long-term complications, including the need for prosthetic joint replacement.

### 4.3 Challenges and strategies for antibiotic therapy in septic arthritis

Antibiotic therapy remains the cornerstone of treatment for septic arthritis. However, the increasing resistance to antibiotics brings great challenges to traditional antibiotic therapy. While the prevalence of healthcare-associated (HA) MRSA has declined in some settings, community-associated (CA) MRSA clones have become increasingly predominant in both community and healthcare environments (Brahami et al., 2025). Furthermore, the expansion of methicillin-susceptible *S. aureus* (MSSA) lineages, particularly those with enhanced virulence or toxin production, further complicates the treatment landscape and requires ongoing surveillance (Asantewaa et al., 2025). Additionally, a previous study has shown that even antibiotic-killed *S. aureus* can continue to provoke destructive arthritis in a mouse model (Ali et al., 2015b), stressing the need for innovative therapeutic strategies.

Therefore, new antibiotic strategies and combination therapies are being explored. First, the use of multiple antibiotic therapies has shown effectiveness in cobating drug-resistant strains. For example, linezolid in combination with other antibiotics can increase the therapeutic effect against MRSA infection, as reviewed in Chen et al. (2020). Also, studies indicate that combining antibiotics with antibiofilm agents can more effectively penetrate and remove bacterial biofilms within joints (Jacqueline and Caillon, 2014).

However, the challenges of antibiotic therapy extend beyond drug resistance. Issues like drug permeability and toxicity in joint tissues also need to be addressed. To increase the effective concentration of the drug in the articular space while reducing systemic toxicity, local drug delivery systems like hydrogels, nanoparticles, and microspheres have been investigated to deliver the drug directly to the site of infection (Ghazagh and Frounchi, 2024; Ahmad et al., 2024; Hsu et al., 2018). These novel delivery systems can create a reservoir of drugs in the joint cavity, allowing antibiotics to be maintained locally at higher concentrations, thereby improving treatment efficacy.

Moreover, novel therapeutic approaches are also being explored. One such approach is the use of antimicrobial peptides (AMPs). These mostly cationic peptides are known for their rapid bactericidal action and effectiveness against a wide range of drug-resistant pathogens. AMPs represent a promising class of antibacterial agents with potent antimicrobial activity against various pathogenic microorganisms. However, their efficacy is challenged by bacterial resistance mechanisms, particularly those of *S. aureus*, which can actively reduce its net negative surface charge through MprF-mediated lysinylation of phosphatidylglycerol and DltABCD-dependent alanylation of teichoic acids, thereby repelling cationic AMPs. Strategies to overcome this, such as developing AMP analogs resistant to charge repulsion or combining AMPs with resistance pathway inhibitors, are under investigation (Ernst and Peschel, 2011; Weidenmaier et al., 2005). Notably, AMPs have demonstrated synergistic effects with conventional antibiotics (Reffuveille et al., 2014; Lewies et al., 2019), supporting their potential as adjunct therapy for septic arthritis and other bacterial infections.

Another promising avenue involves the use of lytic bacteriophages, commonly known as phage therapy, to target and kill pathogenic bacteria. Phage therapy has been tested against bone and joint infections, including rabbit *S. aureus* osteomyelitis and rat *S. aureus* prosthetic implant infections (Morris et al., 2019; Kishor et al., 2016). However, studies specifically focused on the use of phages to treat acute septic arthritis are still lacking.

## 5 Concluding remarks

The role of different virulence factors of *S. aureus* significantly impacts the host's immune response, influencing the severity of infections such as septic arthritis and bacteremia. This impact is particularly influenced by factors such as aging and TLR2, as demonstrated by the findings in this review.

Animal models have identified pivotal virulence determinants: surface adhesins (vWbp, ClfA, SpA) mediate joint invasion by anchoring to host matrices, while Lpps trigger destructive inflammation. The studies further reveal that PSMs exhibit divergent roles: PSMα exacerbates systemic infection by suppressing neutrophil NADPH oxidase activity and impeding bacterial clearance, whereas PSMβ attenuates joint inflammation through immune modulation, protecting against tissue damage.

Host immunity critically shapes outcomes. Neutrophils and complement (notably C3) constitute essential bloodborne defenses that prevent joint invasion. Monocytes and macrophages paradoxically drive bone erosion despite their bactericidal functions, while adaptive immunity (B/T cells) plays incompletely defined roles in animal models. Cytokine networks significantly influence disease progression, with aging and TLR2 hyperactivation amplifying RANKL-mediated osteoclastogenesis and bone destruction.

Innovative therapeutic strategies emerge from these insights. While anti-virulence vaccines targeting key factors (e.g., vWbp, ClfA, SpA) hold promise, none of these currently exist, particularly those targeting single virulence factors (Caldera et al., 2024; McNeely et al., 2014; Schaffer and Lee, 2009). Importantly, adjuvant osteoprotection approaches, combining antibiotics with anti-RANKL biologics to suppress osteoclast-mediated bone resorption, have demonstrated superior efficacy compared to antibiotic treatment alone in mouse models. Together, these strategies may offer hope for preventing joint destruction and functional impairment.

**Figure 7:**
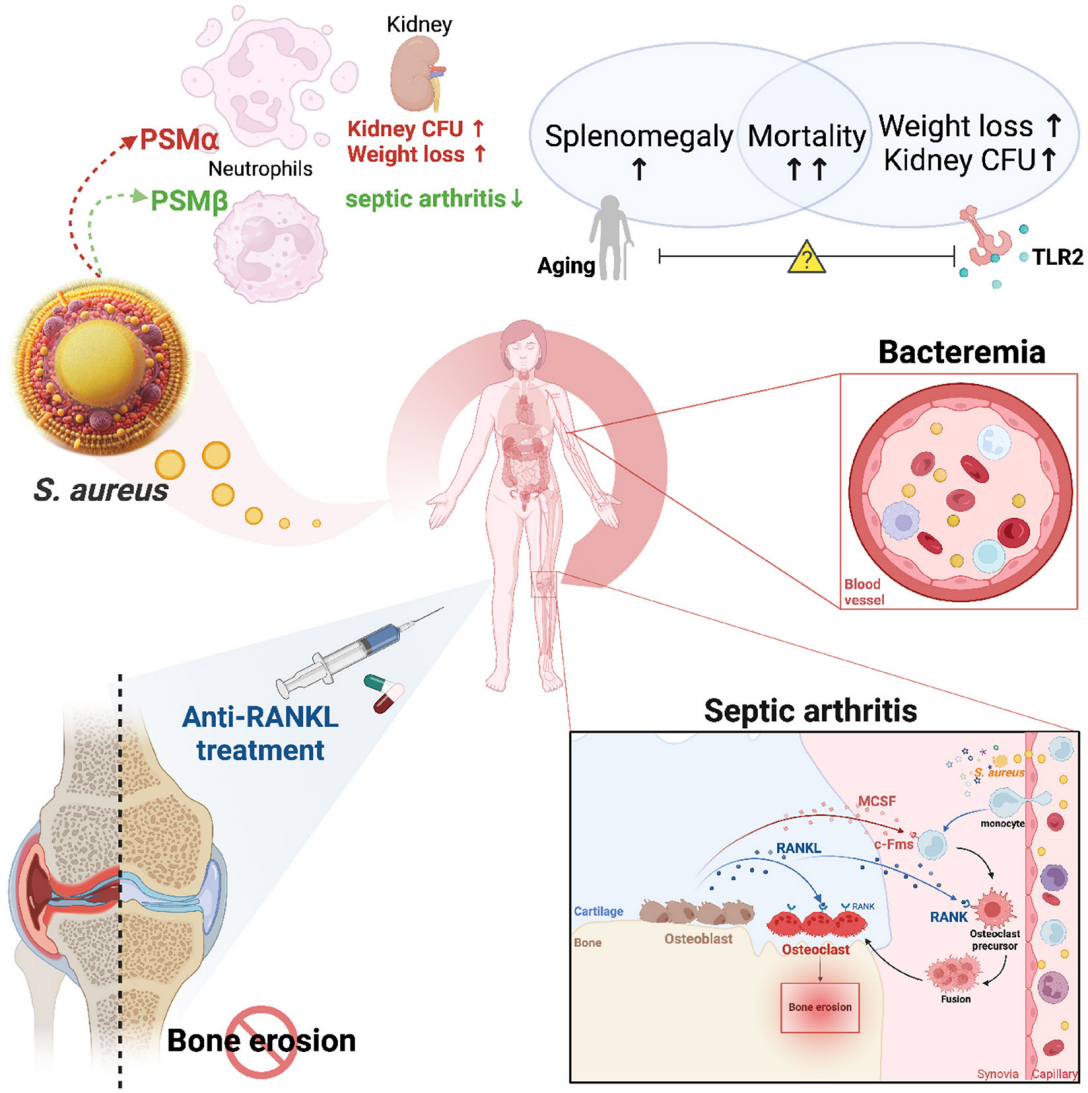
Summary of key findings in this article. This figure illustrates the complex interplay between bacterial virulence factors, host immune responses, and therapeutic interventions in the context of Staphylococcus aureus-induced septic arthritis. Bacterial virulence factors: *S. aureus* secretes various virulence factors, including phenol-soluble modulins (PSMα and PSMβ), which play crucial roles in disease progression. PSMα weakens innate immunity, causing higher weight loss and increased bacterial load (colony-forming units, CFUs) in the kidneys. Conversely, PSMβ is associated with protection against septic arthritis by neutralizing PSMα-induced immune responses. Host factors and immune responses: Host factors such as aging and Toll-like receptor 2 (TLR2) deficiency exacerbate the severity of bacteremia. These factors lead to increased mortality, splenomegaly, and enhanced susceptibility to *S. aureus* infection. Bone Erosion in Septic Arthritis: The figure emphasizes the osteoclastogenesis process within the joint environment, highlighting the role of the RANK/RANKL signaling pathway. After dissemination from the bloodstream, *S. aureus* attracts monocytes to migrate into the inflamed synovial tissue. RANKL (Receptor Activator of Nuclear Factor Kappa-*B* Ligand), produced by osteoblasts and synovial fibroblasts, drives the differentiation of osteoclast precursors into mature osteoclasts, leading to focal bone erosion seen in septic arthritis. Therapeutic interventions: The application of anti-RANKL treatment is shown to inhibit osteoclastogenesis, thereby preventing bone erosion in septic arthritis. The figure illustrates how targeted therapies can mitigate bone damage, emphasizing the importance of RANKL as a therapeutic target in preventing the long-term consequences of septic arthritis.

The complexity of *S. aureus* infections demands context-specific solutions. As illustrated in Figure 7, future success will depend on stratified approaches that target both pathogen virulence and host vulnerability, with particular focus on aging populations, to alleviate the global burden of septic arthritis.
